# Precise Epitaxial 1D and 2D Growth of Polyester‐Based Materials in *n*‐Alkanes

**DOI:** 10.1002/chem.202501290

**Published:** 2025-05-24

**Authors:** Simon D. Dale, Megan R. Elliott, Arianna Brandolese, Andrew P. Dove, Rachel K. O'Reilly

**Affiliations:** ^1^ School of Chemistry University of Birmingham Edgbaston Birmingham B15 2TT UK

**Keywords:** crystallization‐driven self‐assembly, cylinder, living crystallization‐driven self‐assembly, nanoparticle, platelet

## Abstract

Crystallization‐driven self‐assembly (CDSA) has been extensively studied for the formation of bespoke nanoparticles and provides a unique way to control the unidirectional growth of block copolymers (BCPs). Currently, oil‐soluble nanoparticles represent an under‐researched area in the literature, stemming from the difficulty in synthesizing organic nanoparticles with higher‐order morphologies using traditional techniques. These oil‐soluble nanoparticles have uses as components in products as diverse as electronics and engine oils, with current research determining a strong relationship between morphology and performance, with anisotropic nanoparticles outperforming spherical counterparts. Here, we report on the facile self‐assembly of polyester‐based BCPs in *n*‐octane to achieve low‐dispersity 1D and 2D nanoparticles. This report focuses on using tunable, oil‐soluble polymers and aims to understand their self‐assembly in *n*‐octane through the variation of self‐assembly conditions and unimer solubility to form nanoparticles of a controlled and variable size.

## Graphical abstract

Living crystallization‐driven self‐assembly is a facile technique for the synthesis of low‐length dispersity 1D and 2D nanoparticles. Oil‐based systems have applications in electronics and as friction modifiers and are underreported in literature. In this work, we synthesize tunable block copolymers with a poly(ε‐caprolactone) (PCL) core‐forming block and poly(n‐methacrylate) corona block. We report on their self‐assembly in n‐octane for the synthesis of monodisperse nanoparticles of a controlled length and morphology.

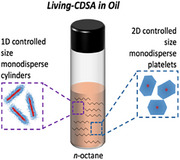

## 1 Introduction

Solution‐state self‐assembly of block copolymers (BCPs) has recently been extensively studied to synthesize bespoke nanoparticles.^[^ ^1^, ^2^, ^3^ ^]^ Current techniques to form nanoparticles tend to yield spherical morphologies as a consequence of the synthetic procedures and applied, with spheres often being the lowest‐energy morphology.^[^ ^4^, ^5^, ^6^ ^]^ Anisotropic nanoparticles, however, are of interest, arising from their comparatively improved performance in drug delivery systems, increased thermoresponsive behavior, and other areas.^[^ ^7^, ^8^ ^]^

The formation of these 1D and 2D particles relies on specific conditions that exist in a small phase space.^[^ ^6^ ^]^ Crystallization‐driven self‐assembly (CDSA) is a facile entry point for these hard‐to‐achieve anisotropic morphologies.^[^ ^9^ ^]^ In CDSA, anisotropic nanoparticles are formed through the crystallization of the core‐forming block as the crystalline packing of the polymer core favours a lower‐interfacial curvature, exclusively leading to cylinders and platelets.^[^ ^4^, ^5^, ^10^ ^]^ CDSA can also be exploited to create monodisperse 1D and 2D nanoparticles of a controlled size. This process, known as living‐CDSA (L‐CDSA), exploits the kinetic and thermodynamic process of crystallization for the formation of monodisperse nanoparticles.^[^ ^11^ ^]^ Thanks to its ability to create reliable monodisperse particles through simple sample preparation, living‐CDSA is the focus of much current CDSA research.^[^ ^3^, ^12^, ^13^, ^14^, ^15^, ^16^ ^]^

In CDSA, the core‐forming block plays a significant role as it drives the crystallization process.^[^ ^17^, ^18^ ^]^ Polyester‐based core‐forming blocks have been widely studied,^[^ ^19^ ^]^ including poly(ɛ‐caprolactone) (PCL). Understanding how PCL crystallizes in these systems has allowed for a wide range of coronal chemistries to be investigated and the development of polymers that are applicable for a variety of uses.^[^ ^20^, ^21^, ^22^ ^]^ However, much of this work has focused on water‐based systems as a consequence of the biocompatibility and biodegradability of PCL and interest in designing materials for delivery and tissue engineering end applications.^[^ ^14^, ^21^, ^23^, ^24^, ^25^ ^]^

Forming oil‐soluble nanoparticles is an important area of study as a consequence of their potential use in emulsions, engine oils, and electronics. Emerging studies have found that oil‐dispersed nanoparticles can display improved performance in these areas.^[^ ^26^, ^27^, ^28^, ^29^, ^30^ ^]^ Notably, morphological effects have been reported to have an impact on performance, especially in oil‐based systems. For example, anisotropic particles have been shown to have unique material properties, such as the ability to orient under shear flow, significantly decreasing friction and wear,^[^ ^31^, ^32^, ^33^ ^]^ and platelet particles have increased heat capacity owing to their large surface area.^[^ ^34^ ^]^ Many of these studies have indicated that particles with higher‐order morphologies have a positive impact on performance in a variety of applications, namely engines, where they are able to decrease friction, greatly increasing the lifetime.^[^ ^35^ ^]^ Notably, these studies remain mainly limited to the use of inorganic nanoparticles, likely a consequence of the challenges of synthesizing organic particles with controlled dimensions.

The ability to control nanoparticle shape, size and surface chemistry is of great importance to understand the impacts of these variables on the performance of such nanoparticles in advanced materials applications. The application of CDSA to create anisotropic particles with controlled dimensions and functional group placement represents an interesting area to advance for use in oil‐based systems.^[^ ^31^, ^36^, ^37^, ^38^ ^]^

Herein, we report the application of PCL‐based diblock copolymers in CDSA in *n*‐alkanes. PCL was used as the core‐forming block, to create particles that would enable the nature of the corona‐forming block on CDSA behavior to be understood. The growth of the particles in *n*‐octane was achieved by two approaches that were focused on controlling the solubility of the unimers to enable living CDSA to occur. First, variation of the growth conditions, including temperature and solvent composition, was undertaken, with the second approach examining the chemical structure of the polymethacrylate corona‐forming block. Both 1D and 2D growth were successful and were able to be applied to achieve particles with predictable shapes and dimensions through facile changes of seed‐to‐unimer ratios.

## 2 Results and Discussion

### 2.1 Block Copolymer Synthesis

Block copolymers (BCPs) were synthesized through two subsequent polymerizations. A ring‐opening polymerization (ROP) of ɛ‐caprolactone was first performed using a dual‐headed chain‐transfer agent (CTA) as the initiator to create macro‐CTA poly(ɛ‐caprolactone) (PCL) at a degree of polymerization (DP) of 50 (Figures S1–S4, Supporting Information). The macro‐CTA was used for reversible‐addition fragmentation chain‐transfer (RAFT) polymerization to create a library of BCPs at DPs of 60 and 155. RAFT was employed as a consequence of its facile ability to make BCPs with controlled number average molecular weight (*M*_n_) (Table S1, Supporting Information). This work used three monomers, lauryl methacrylate (LMA), *n*‐octyl methacrylate (*n*OMA), and *n*‐decyl methacrylate (*n*DMA), to create the corona block, an oil‐soluble unit, of the BCPs: PCL_50_‐*b*‐PLMA_150_, PCL_50_‐*b*‐PLMA_60_, PCL_50_‐*b*‐P*n*OMA_148_, and PCL_50_‐*b*‐P*n*DMA_155_. Previously, homopolymers of these monomers have shown good solubility in oils, and the monomers are readily available.^[^ ^39^ ^]^ A DP of ≈150 of the corona unit was chosen to create diblock copolymers with a 1:3 core:corona ratio. For PCL_50_‐*b*‐PLMA_60_ a DP of 60 was chosen to investigate the effects of a lower core:corona ratio and decrease the solubility of the solvophillic unit. All diblock copolymers were able to undergo self‐nucleation to form polydisperse anisotropic nanoparticles, and the solution‐state differential scanning calorimetry (nano‐DSC) was consistent with PCL being the core‐forming block (Figures S21 and S22, Supporting Information).^[^ ^23^, ^26^, ^27^, ^40^ ^]^

### 2.2 1D Epitaxial Growth

To form low length‐dispersity seed particles, polydisperse cylinders of PCL_50_‐*b*‐PLMA_150_ formed via CDSA were sonicated in an ice bath for 1 h 40 min and had an average length (*L*_n(seed)_) of 41 nm, as determined by transmission electron microscopy (TEM) (Figure S20, Supporting Information). Sonication was able to effectively break down the cylinders into seed particles, which acted as the starting point for the growth of anisotropic semi‐crystalline nanoparticles. The as‐formed seed particles were used for all the following studies.

To better understand the effect of unimer solubility on living‐CDSA, the epitaxial growth onto seed particles was tested. This was studied by varying unimer‐to‐seed ratios (*m*_unimer_ */m*_seed_). The unimer‐to‐seed ratio was altered by changing the concentration of diblock copolymer solution to keep the ratio of bad solvent to good solvent consistent across samples. TEM was used to analyse length dispersity of *m*_unimer_ */m*_seed_ of 1, 10, and 25. The theoretical length was calculated using Equation (1):

(1) $$L_{\mathrm{theory}}=\left(L_{n(\mathrm{seed})}\times \left(m_{\mathrm{unimer}}/m_{\mathrm{seed}}\right)\right)+L_{n(\mathrm{seed})}$$

where *L*_theory_ is the theoretical length of the cylinders based on their unimer‐to‐seed ratio and *L*_n(seed)_ is the average length of the seeds, calculated through analysis of TEM micrographs (Figure S20, Supporting Information).

**Figure 1:**
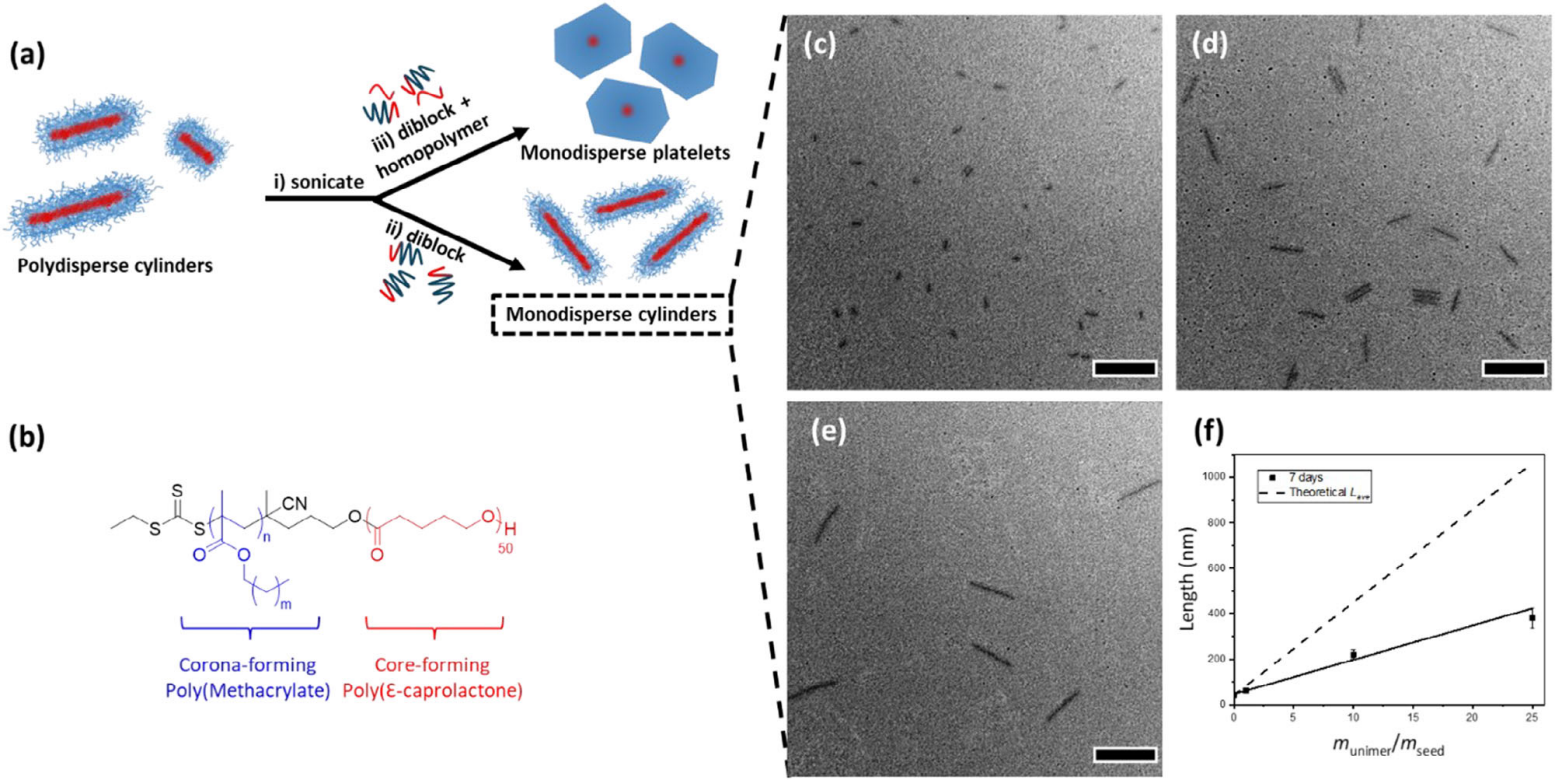
a) Overview of living‐CDSA process. b) Chemical structures of diblock copolymers. c–e) TEM micrographs of living‐CDSA solutions of PCL_50_‐*b*‐PLMA_150_ after 7 days of aging at room temperature with *m*_unimer_/*m*_seed_ ratios (c) 1, (d) 10, and (e) 25. f) Plot showing the linear relationship between different *m*_unimer_/*m*_seed_ ratios with narrow length dispersity (error bars represent standard deviation), compared to the theoretically expected cylinder length. Scale bars = 500 nm.

After preparation and aging for 7 days at room temperature, the formed cylinders were much shorter than expected from the predicted length (*L*_theory_) (Figure 1). For a unimer to seed ratio of 10, the *L*_theory_ was calculated to be 451 nm, whereas experimentally, after 7 days the cylinders were measured to be 220 ± 21 nm (Table S2, Supporting Information). However, despite this limitation, analysis of the TEM micrographs at three different seed‐to‐unimer ratios enabled the successful observation of epitaxial growth, as indicated by a linear increase in cylinder length with increasing seed‐to‐unimer ratios, while retaining a narrow length dispersity (Figure 1). The shorter than expected cylinders suggested that the unimers were highly soluble in the assembly media, as there was no evidence of self‐nucleation in the TEM analysis, but the observed monodisperse cylinders were shorter than the predicted length. This indicates that the unimers were still in solution but not participating in growth. The solutions were left to age for 56 days, after which time the experimentally observed cylinder length increased to 303 ± 42 nm. While the cylinders were significantly longer than before aging, they were still shorter than the theoretical length based on the seed‐to‐unimer ratios. The cylinders also increased in length dispersity, likely a result of self‐nucleation of the dissolved unimer. Self‐nucleation occurs when individual unimers undergo crystallization and act as a growth point, leading to polydisperse samples. These observations indicate two possibilities: that the energy barrier for crystallization was too high to overcome, or that the rate of crystallization was too slow. Both routes would lead to large amounts of dissolved unimer in solution.

In order to avoid self‐nucleation and achieve the synthesis of cylinders with predictable lengths, we focused on increasing the speed of crystallization of the core‐forming block. To achieve this, we first lowered the aging temperature, which has been previously reported to improve the living growth of PFS‐based systems, owing to the impact of supercooling on the crystal growth.^[^ ^41^ ^]^ To this end, the temperature was decreased to 4 °C by aging in a fridge. Unexpectedly, after aging for 35 days, the average cylinder length had decreased for all *m*_seed_ */m*_unimer_ ratios, with *m*_seed_/*m*_unimer_ = 10 decreasing from 278 ± 34 to 184 ± 44 nm (Tables S2 and S3, Supporting Information). The reduction in length seen from the lower cylinder length is attributed to the lower temperature increasing the energy barrier for crystallization, leading to less material crystallizing. A cosolvent was then employed for further studies to attempt to speed up the crystalline growth. *n*‐Heptane was used as the cosolvent as its shorter chain length reduces the solubility of the corona thus disfavouring the solubility of the unimer in solution. Varying percent volumes of *n*‐heptane in *n*‐octane were investigated from 1 to 50%v/v with cylinder length imaged after 7 days (Figure S23 and Table S4, Supporting Information). An increase in length was observed at and above the addition of 20%v/v volume of *n*‐heptane, showing the highest 7‐day length occurring at 40%v/v *n*‐heptane with an average cylinder length of 285 ± 48 nm. This solution was aged for a further 22 days by which time the cylinders were observed to be 374 ± 48 nm by TEM analysis. These conditions yielded cylinders longer than were obtained in 100% *n*‐octane (278 ± 34 nm) but were still lower than the predicted length of 451 nm (Figures S24 and S25 and Table S5, Supporting Information). The octane/heptane solution was aged for a further 14 days and reimaged, but the calculated length remained the same at 376 ± 46 nm. This indicated that the unimer was still too soluble, even in the presence of a cosolvent.

As the conditions could not be altered to increase the speed of growth, the focus shifted to the polymer itself in order to reduce its solubility in the assembly media. In this framework, the DP of the solvophilic block was lowered to reduce the solubility of the corona. To this end, PCL_50_‐*b*‐PLMA_60_ was investigated in epitaxial growth from the previously prepared seeds. Five *m*_unimer_/*m*_seed_ ratios were studied. Ratios of *m*_unimer_/*m*_seed_ = 1, 3, 7, 10, and 25 were used to prepare cylinders and were left to age for 7 days (Figure S26, Supporting Information). The cylinders were imaged by TEM, which showed that the lower unimer/seed ratios, ≤10, led to an observed linear relationship that correlated cylinder length to the amount of unimer added to the solution. These lower ratios gave anisotropic nanoparticles of predictable length. Unimer/seed ratios greater than 10 led to regions of shorter cylinders visible in the transmission electron microscopy micrograph (Table S6, Supporting Information). The shorter cylinders indicated that the unimer underwent self‐nucleation, which could have arisen from the high concentration of unimer in solution. To overcome the high concentration of unimer, the synthesis of cylinders with *m*_unimer_/*m*_seed_ = 25 was attempted through five sequential additions of unimer (Figure S27, Supporting Information). Sequential additions were hypothesized to lower the amount of free unimer in solution, lessening the chance of self‐nucleation. After three additions, self‐nucleation was still present, most likely arising from increased unimer concentration over time. This indicated that the solubility of the corona was still too high, and hence, other corona chemistries were investigated.

In preference to further iterating polymer design, octanol‐water partition coefficients (LogP_oct_) were examined to guide corona choice. LogP_oct_ is a common technique used to study the hydrophobicity of molecules, with LogP_oct_ over surface area (LogP_oct_/SA) having being utilised to understand the hydrophobicity of macromolecular systems.^[^ ^42^ ^]^ Calculations for *n*‐octane have shown that LogP_oct_/SA = 0.022. A poly(*n‐*decyl methacrylate) (P*n*DMA) oligomer proxy was calculated to have a LogP_oct_/SA of 0.023, while a poly(*n*‐octyl methacrylate) (P*n*OMA) oligomer had a value of 0.021, both of which sit below PLMA, which is 0.024.^[^ ^23^ ^]^ This indicated that these monomers have a better match of solubility to *n*‐octane, which we anticipated would give better control over the crystallization process.

CDSA of PCL_50_‐*b*‐P*n*DMA_155_ and PCL_50_‐*b*‐P*n*OMA_146_ BCPs was undertaken through self‐nucleation studies in *n*‐octane at 5 mg mL^−1^ to determine if the BCPs were able to undergo crystalline growth (Figure S19, Supporting Information). Nano‐differential scanning calorimetry (nano‐DSC) analysis of the resulting solution revealed the appearance of a melting peak, which indicated that the material had crystallized over the 2 week aging period. Additional analysis of the nanoparticle‐containing solution by dynamic light scattering (DLS) and TEM showed the appearance of nanoparticles.

**Figure 2:**
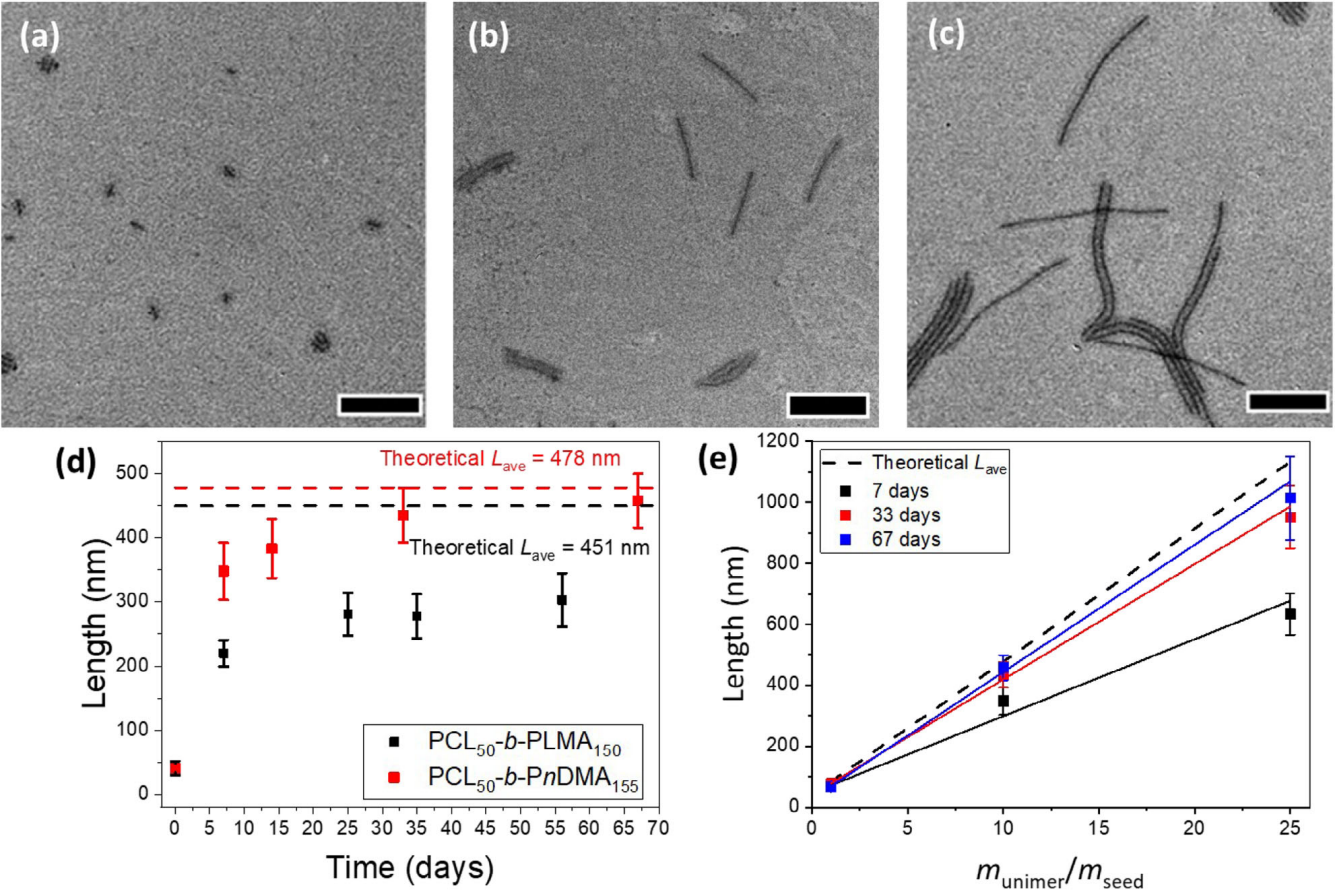
a–c) TEM micrographs of 1D epitaxial growth of PCL_50_‐*b*‐P*n*DMA_155_ from seeds of PCL_50_‐*b*‐PLMA_150_ in a *m*_unimer_/*m*_seed_ ratio of (a) 1, (b) 10, and (c) 25 after 67 days. Scale bars = 500 nm. d) Plot showing the linear relationship between different *m*_unimer_/*m*_seed_ ratios with narrow length dispersity after 7, 33, and 67 days (error bars represent standard deviation), compared to the theoretically expected cylinder length. e) Comparison of cylinder lengths of PCL_50_‐*b*‐PLMA_150_ and PCL_50_‐*b*‐PnDMA_155_ grown from seeds of PCL_50_‐*b*‐PLMA_150_ at a *m*_unimer_/*m*_seed_ ratio of 10 over time.

To compare the living CDSA behavior of these polymers, a *m*_unimer_/*m*_seed_ = 10 was used to study their epitaxial growth onto the previously prepared seed micelles. The nanoparticles from PCL_50_‐*b*‐P*n*OMA_146_ had a broad cylinder length distribution arising from the greatly reduced solubility of the shorter alkyl side chain in *n*‐octane (Figure S28, Supporting Information). In contrast, CDSA using PCL_50_‐*b*‐P*n*DMA_155_ yielded low length‐dispersity cylinders that agreed with the calculated *L*_theory,_ indicating that this polymer corona led to good control over solubility and, in turn, crystallization (Figure 2d).

After aging for 7 days, the cylinders were smaller than the calculated *L*_theory_, with the observed length = 348 ± 46 nm when *m*_unimer_ */m*_seed_ = 10 (*L*_theory_ was calculated to be 478). The sample was monodispersed. After further aging for 33 and 67 days, the cylinders measured to be 435 ± 42 and 458 ± 42 nm, respectively, in much closer agreement to the lengths predicted from the unimer/seed ratio (Figure 2e). Unlike in the PCL_50_‐*b*‐PLMA_150,_ aging for longer periods did not lead to increased self‐nucleation, which indicated that the reduction in solubility of the P*n*DMA corona decreased the barrier to crystallization and enabled all unimers to assemble into 1D nanoparticles. This highlighted that PCL_50_‐*b*‐P*n*DMA_155_ represented a “middle ground,” allowing for controlled growth that avoided self‐nucleation.

### 2.3 Controlled 2D Epitaxial Growth

Living‐CDSA is also widely used to form 2D nanoparticles.^[^ ^16^, ^23^, ^24^, ^43^ ^]^ 2D platelets are commonly achieved through co‐crystallization with homopolymer acting as a point of crystal growth. In order to better understand the living‐CDSA of PCL core‐based systems in oil, we focused our attention on their 2D‐expitaxial growth.

To gain insight into the effects of additional homopolymer on the self‐assembly of PCL_50_‐*b*‐PLMA_60_ in *n*‐octane, mass ratios of homopolymer/diblock (*m*_PCL50_/*m*_diblock_) were varied and *m*_unimer_/*m*_seed_ ratio was kept constant at 10. The homopolymer/diblock ratio, *m*_PCL50_/*m*_diblock_, was altered by changing the amount of PCL_50_ dissolved in a 10 mg mL^−1^ unimer solution. This new unimer solution was added to the previously formed seeds.

First, PCL_50_‐*b*‐PLMA_60_ was investigated as PLMA has been shown in other systems to have increased performance in oil‐based systems and core/corona ratio of 1 often gives 2D morphologies.^[^ ^38^, ^44^ ^]^ Ratios of *m*_PCL50_/*m*_diblock_ <1 resulted in no observed effect on particle morphology; only cylinders were formed (Figure S30, Supporting Information). This is likely a consequence of the low levels of semi‐crystalline homopolymer failing to drive crystallization onto the crystalline platelets. An *m*_PCL50_/*m*_diblock_ ratio of greater than 1 led to platelet‐like particles observed in the TEM micrographs (Figure S30c, Supporting Information). The observed platelets were polydisperse, which indicated that the crystallization of the diblock copolymer onto the homopolymer was not controlled, likely as a consequence of low unimer solubility or homopolymer self‐nucleation.

**Figure 3:**
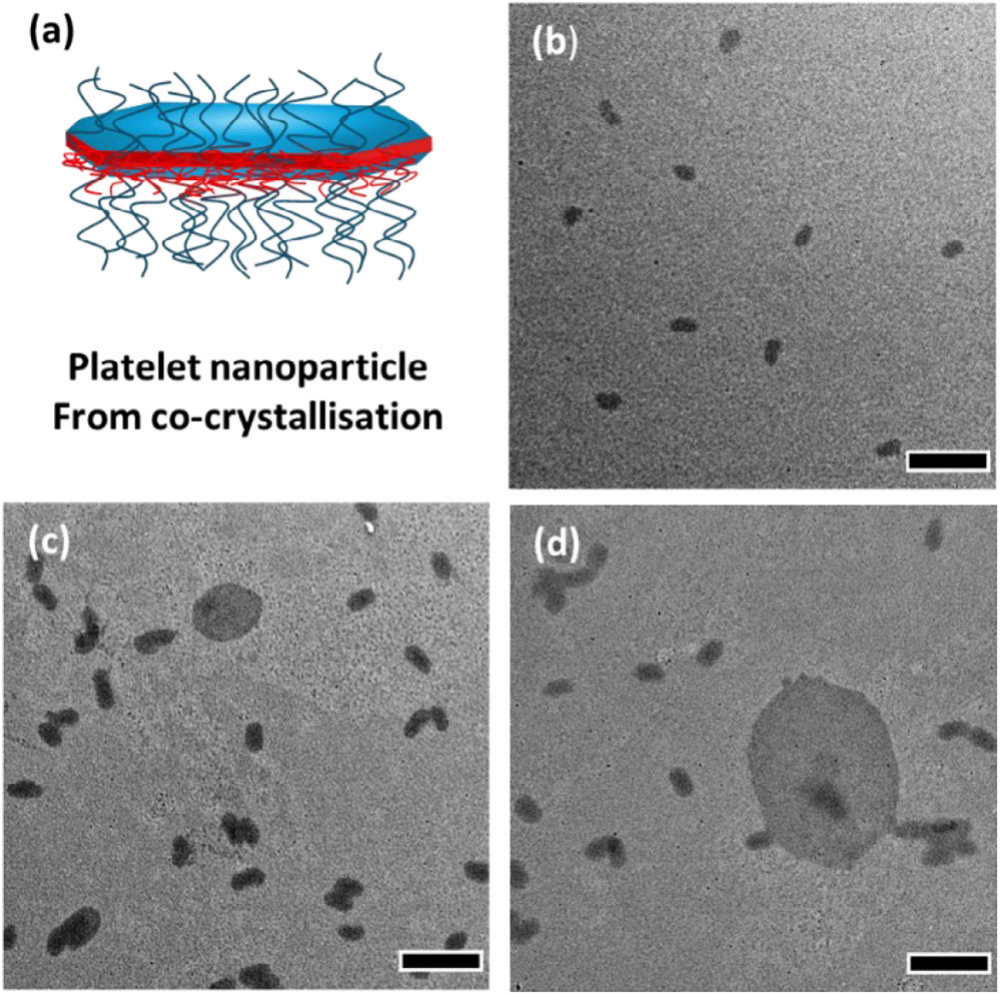
a) Overview of morphology of 2D platelet from co‐crystallization of PCL_50_. b–d) TEM micrographs of a living growth solution of PCL_50_‐*b*‐PLMA_150_ unimer co‐crystallized with PCL_50_ homopolymer onto seeds of PCL_50_‐*b*‐PLMA_150_ where *m*_unimer_/*m*_seeds_ = 10 and *m*_PCL50_/*m*_unimer_ = 1 after (b) 7, (c) 25, and (d) 135 days. Scale bar = 500 nm.

Previous work in the group had found that for polyester‐based systems, higher core/corona ratios led to the successful formation of 2D morphologies.^[^ ^45^ ^]^ PCL_50_‐*b*‐PLMA_150_ was therefore employed as it had a 3/1 core/corona ratio and an LMA solubilizing block. This BCP demonstrated the successful formation of monodisperse platelet particles after aging for 7 days (Figure 3b)

To determine if the platelets were stable, they were imaged again after 35 days of aging (Figure 3c), which revealed large platelets. The solutions were then left to age again for a total of 135 days before being reimaged (Figure 3d). Over the increased time, the platelets demonstrated increased growth of width relative to length, determined by the decrease in Length/Width (*L*_n_/*W*_n_) of 1.84 to 1.75 after 35 and 135 days, respectively (Table S10, Supporting Information). The formation of larger platelets was likely due to PCL_50_ homopolymer self‐nucleating and crystallizing at a faster rate than the rate of diblock copolymer crystallization onto the seeds. This again pointed to the high solubility of PCL_50_‐*b*‐PLMA_150_ in *n*‐octane.

**Figure 4:**
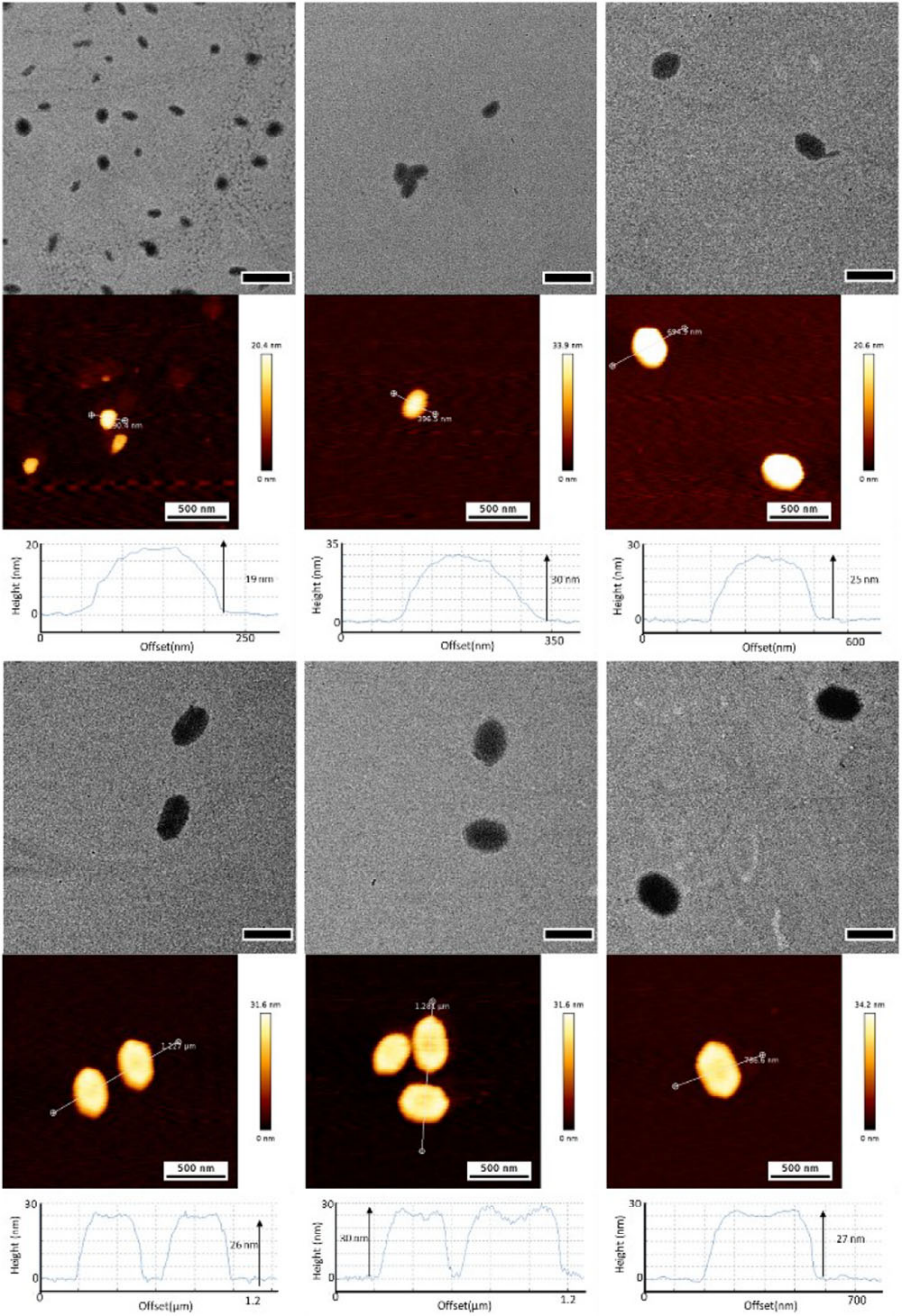
TEM and AFM micrographs with cross‐section height measurements of platelet nanoparticles of PCL_50_ and PCL_50_‐*b*‐P*n*DMA_155_ co‐crystallized from seeds of PCL_50_‐*b*‐PLMA_150_ in *m*_unimer_/*m*_seed_ ratios (top row) 1, 10, 25, (bottom row) 40, 60, and 100 (from left to right). Scale bars = 500 nm.

As a consequence of the slow unimer crystallization, PCL_50_‐*b*‐P*n*DMA_155_ was employed as the shorter chain length was anticipated to reduce the solubility of the unimers. A *m*_PCL50_/*m*_unimer_ ratio of 1 was used, and *m*_unimer_/*m*_seeds_ was varied, studying ratios of 1, 10, 25, 40, 60, and 100. The solutions were analysed after 60 days to allow for sufficient growth time. The targeted platelet morphology was obtained by co‐crystallization of PCL_50_ and PCL_50_‐*b*‐P*n*DMA_155_, and the resulting nanoparticles were characterised by TEM and atomic force microscopy (AFM). For *m*_unimer_/*m*_seeds_ up to 40, there was a linear increase in platelet length and width, which is characteristic of living‐CDSA systems (Figure 4). As the *m*_unimer_/*m*_seeds_ ratio increased above 40, the *L*_n_/*W_n_* converged to ∼1.5 (Table S12, Supporting Information). Interestingly, as the unimer‐to‐seed ratio was increased from 1 to 40, the *L*_n_/*W_n_* decreased from 2 to 1.56, indicating that the width of the platelets was increasing at a greater rate than the length of the platelets. The observed decrease in the *L*_n_/*W*_n_ demonstrated a preference for the unimer/homopolymer blend to crystallize perpendicular to the seed axis. The *L*_n_/*W*_n_ of the platelets of PCL_50_‐*b*‐P*n*DMA_155_ is larger than those assembled using PCL_50_‐*b*‐PLMA_150_ at the same *m*_unimer_/*m*_seeds_ ratios. At a *m*_unimer_/*m*_seeds_ = 10, the PCL_50_‐*b*‐P*n*DMA_155_ *L*_n_/*W*_n_ = 1.90 whereas for PCL_50_‐*b*‐PLMA_150_ *L*_n_/*W*_n_ = 1.75. The higher *L*_n_/*W*_n_ could arise as a consequence of the lower solubility, leading to a greater amount of unimer crystallizing onto the nanoparticles. PCL_50_‐*b*‐P*n*DMA_155_ was able to undergo controlled 2D epitaxial growth as a consequence of the tuned solubility of the *n*‐decyl methacrylate side chain in *n*‐octane. These platelet's size can be controlled by unimer addition to obtain increasing *L*_n_/*W*_n_.

## 3 Conclusion

Synthesis of monodisperse anisotropic cylinders in *n*‐alkanes has been under‐studied in current CDSA literature. In this work, the living‐CDSA of polyester‐based BCPs in *n*‐alkanes was investigated. We were able to demonstrate the successful synthesis of easily modifiable poly(ɛ‐caprolactone)‐containing diblock copolymers that are able to undergo 1D and 2D epitaxial crystalline growth. This growth allowed for the formation of both 1D and 2D nanoparticles of a controlled and modifiable length through facile self‐assembly methods. These were able to be fully performed in *n*‐alkanes, demonstrating that partially (bio)degradable materials are applicable for oil‐based applications, helping to create more sustainable alternatives. Finally, we were able to study the effects of solvent composition, temperature, and unimer solubility on the living growth of these nanoparticles and used LogP/SA analysis to guide our choice of coronal chemistries. We envisage that future directions of this work could lead to the use of monodisperse anisotropic organic nanoparticles in a variety of oil‐soluble systems, including anti‐wear agents, friction modifiers, and emulsifiers. This proof‐of‐concept would enable researchers to study size and/or morphological correlations of organic nanoparticles and their performance in these systems.

## Supporting Information

The authors have cited additional references within the Supporting Information.

## Conflict of Interests

The authors declare no conflict of interest.

## Supporting information



Supporting Information

## Data Availability

The data that support the findings of this study are available in the supplementary material of this article.
