# Supplementary material for: Precise Epitaxial 1D and 2D Growth of Polyester‐Based Materials in n‐Alkanes
Source: Chemistry. 2025 May 24;31(35):e202501290. doi: 10.1002/chem.202501290 (PMC12188167; doi:10.1002/chem.202501290)
Supplement: Supplementary file 1 — Supporting Information [file CHEM-31-e202501290-s001.docx]

Precise Epitaxial 1D and 2D Growth of Polyester-Based Materials in *n*-Alkanes

Simon D. Dale,^[a]^ Megan R. Elliott,^[a]^ Arianna Brandolese,^[a]^ Andrew P. Dove,*^[a]^ and Rachel K. O’Reilly*^[a]^

*^[a]^ School of Chemistry, University of Birmingham, Edgbaston, Birmingham B15 2TT UK*

E-mail: [a.dove@bham.ac.uk](mailto:a.dove@bham.ac.uk) [r.oreilly@bham.ac.uk](mailto:r.oreilly@bham.ac.uk)

**Experimental**

**Materials**

Chemicals were purchased from Merck, Alfa Aesar, VWR, or Generon Ltd. Solvents, lauryl methacrylate, *n*-decyl methacrylate, and octyl methacrylate were purified through basic alumina before use in RAFT polymerizations. AIBN was recrystallised twice from methanol and stored in the dark at 4 °C. Air-free work was conducted in the nitrogen atmosphere of an Mbraun MB Unilab Plus equipped with solvent filter. H_2_O and O_2_ analysers had <0.1 ppm readings throughout the time course of this study.

**Instruments**

*Nuclear Magnetic Resonance (NMR)* spectra were recorded on a Bruker DPX-300 or DPX-400 spectrometer with CDCl_3_ (^1^H NMR *δ* (ppm) = 7.28 (s), ^13^C NMR *δ* (ppm) = 77.72 – 76.42 (t)) as the solvent. Shifts are quoted in *δ* in parts per million.

*Size Exclusion Chromatography (SEC)* measurements were performed in CH_3_Cl on an Agilent 1260 Infinity II Multi-Detector GPC/SEC System fitted with RI, ultraviolet (UV, λ = 309 nm), and viscometer detectors. The polymers were eluted through an Agilent guard column (PLGel 5 μM, 50 × 7.5 mm) and two Agilent mixed-C columns (PLGel 5 μM, 300 × 7.5 mm) using CHCl_3_ (buffered with 0.5% NEt_3_) as the mobile phase (flow rate = 1 mL min^-1^, 40 °C). Number average molecular weights (*M*_n_), weight average molecular weights (*M*_w_) and dispersities (*Đ*_M_ = *M*_w_/*M*_n_) were determined using Agilent GPC/SEC software (vA.02.01) against a 12-point calibration curve (*M*_p_ = 550–2,210,000 g mol^-1^) based on poly(styrene)-medium standards (Easivial PM, Agilent).

*Atomic Force Microscopy (AFM)* was performed on a JPK Nanowizard 4 system at room temperature in the supplied acoustic enclosure and vibration isolation using Nanosensor PPP-NCHAuD tips with a force constant of around 42 N⋅m^-1^. For data acquisition and handling Nanowizard Control and Data Processing Software V.6.1.117 in quantitative imaging (QI) mode with a setpoint of 25 *nN* was used. Samples were prepared on freshly cleaved mica by drop casting 10 µL of assembly solution (0.01 mg mL^-1^) followed by drying in air. The mica disk was then submerged in 2 mL of heptane for 2 minutes to remove residual assembly solvent before again drying in air prior to analysis.

*Transmission Electron Microscopy (TEM)* imaging was performed on a JEOL JEM-1400 microscope operating at an acceleration voltage of 80 kV. Sample of PCL_50_-*b*-PLMA_150_ in octane after 9 months of aging was diluted in octane (0.1 mg mL^-1^) prior to imaging. Living growth experiments with a *m*_unimer_/*m*_seed_ ratio >10 diluted by half prior to imaging. All other samples were imaged without dilution. The sample was drop cast onto formvar-coated copper grids and dried in air. The grids were exposed to ruthenium (VIII) oxide vapour for 7 minutes at 20 °C prior to analysis.^35^ This heavy metal compound acted as a positive stain to improve contrast. The ruthenium(VIII) oxide was prepared as follows: ruthenium(IV) oxide (0.30 g) was added to water (50 g) to form a black slurry. Sodium periodate (2.0 g) was added and stirred for 1 minute prior to use.

*Dynamic light scattering (DLS)* was used to determine hydrodynamic diameters (*D*_h_) and size distributions (PD) of particles using a Malvern Zetasizer Nano ZS equipped with a 4 mW He-Ne 633 nm laser module. Measurements were carried out at an angle of 173° (back scattering), and results were analyzed using Malvern DTS v7.03 software. All determinations were repeated in triplicate with at least 10 measurements recorded for each run. *D*_h_ values were calculated using the Stokes-Einstein equation where particles are assumed to be spherical, while for cylindrical particles DLS was primarily used to detect multiple populations and obtain dispersity information.

*Differential scanning calorimetry (DSC)* was used to determine the thermal characteristics of the polymers using a STARe DSC3 system from Mettler Toledo and analyzed in 40 μL aluminium pans from −100 – 100 °C at a heating rate of 10 °C min^−1^ for two heating/cooling cycles.

*Micro-differential scanning calorimetry (nano-DSC)* was used to measure the crystallisation in the particles. Calorimetric measurements were performed with at a scanning rate of 1 °C min^-1^. The pure solvent was used as a reference. The nano-DSC allows measurements with an extremely high sensitivity using sample masses up to 1 g, hence the detection of phase transitions of polymers in dilute solutions.

**Synthesis**

*Synthesis of chain transfer agent (CTA)/ROP initiator: 2-cyano-5-hydroxypentan-2-yl ethyl carbonotrithioate (CHPET).*

Synthesis of CHPET was conducted following a previously reported method.^1 1^H NMR (400 MHz, CDCl_3_) *δ* (ppm) 3.75 (t, *J* = 6.1 Hz, 2H), 3.36 (q, *J* = 7.4 Hz, 2H), 2.36 – 2.06 (m, 2H), 1.92 (s, 3H), 1.92 - 1.83 (m, 2H), 1.38 (t, *J* = 7.4 Hz, 3H). ^13^C NMR (101 MHz, CDCl_3_) δ = 217.4, 119.6, 61.8, 47.0, 35.8, 31.3, 27.9, 24.9, 12.8.

*Synthesis of a PCL_50_ macro chain transfer agent (CTA)*

In a nitrogen-filled glove box, diphenyl phosphate (0.035 g, 0.140 mmol) in dry toluene (3.5 mL) and CHPET (0.035 g, 0.140 mmol) in dry toluene (6.323 mL) were added to ɛ-caprolactone (1.037 mL, 9.823 mmol). The solution was stirred at room temperature for 6 h and then precipitated into excess diethyl ether at 0 °C for three times and collected *via* Buckner filtration before drying under reduced vacuum over P_2_O_5_ for 2 days (0.807 g).

^1^H NMR (300 MHz, CDCl_3_, 298 K) δ (ppm) 4.05 (t, *J* = 6.7 Hz, 106H), 3.64 (t, *J* = 6.5 Hz, 2H), 3.33 (q, 2H), 2.30 (t, *J* = 7.5 Hz, 106H), 2.13 – 1.91 (m, 2H), 1.88 (s, 3H), 1.73 – 1.56 (m, 218H), 1.46 – 1.30 (m, 110H). SEC (0.5% NEt_3_ in CHCl_3_, λ = 309 nm, PMMA standard) *M*_n_ = 10.5 kg mol^-1^, *Ɖ*_M_ = 1.07.

*Typical RAFT polymerization of methacrylate from PCL_50_ macro chain transfer agent*

PCL_50_ macro-CTA (0.05 g, 0.009 mmol), *n*-decyl methacrylate (filtered through basic alumina, 0.496 g, 2.190 mmol), AIBN (14.40 μL of a 10.00 mg mL^-1^ solution) and toluene (filtered through basic alumina, 1.889 mL) were mixed in a pre-dried ampoule. The homogenous solution was degassed *via* three freeze-pump-thaw cycles and the ampoule back filled with nitrogen. The ampoule was clamped in a pre-heated oil bath at 70 °C for 18 h. Next, the ampoule was removed from the oil bath and held in an ice bath for 10 minutes, whilst exposed to air, before the solution was precipitated into methanol (0 °C) three times. The product was collected and dried *in vacuo*.

^1^H NMR (300 MHz, CDCl_3,_ 298 K) δ (ppm) 4.08 (t, *J* = 6.7 Hz, 106H), 3.95 (s, 316H), 3.67 (t, *J* = 6.4 Hz, 2H), 3.23 (q, *J* = 7.4 Hz, 2H), 2.33 (t, *J* = 7.5 Hz, 106H), 1.92 (s, 84H), 1.82 (s, 99H), 1.74 – 1.53 (m, 710H), 1.47 – 1.24 (m, 1850H), 1.04 (s, 163H), 0.92 (t, *J* = 6.6 Hz, 462H). SEC (0.5% NEt_3_ in CHCl_3_, λ = 309 nm, PMMA standard) *M*_n_ = 30.4 kg mol^-1^, *Ɖ*_M_ = 1.18.

*Characterisation of PCL_50_-b-PLMA_n_*

^1^H NMR (300 MHz, CDCl_3,_ 298 K) δ (ppm) 4.06 (t, *J* = 6.7 Hz, 50H), 3.91 (s, 155H), 2.30 (t, *J* = 7.5 Hz, 50H), 1.84 (d, *J* = 29.8 Hz, 137H), 1.71 – 1.52 (m, 292H), 1.28 (s, 1289H), 1.02 (s, 87H), 0.89 (t, *J* = 6.3 Hz, 406H). SEC (0.5% NEt_3_ in CHCl_3_, λ = 309 nm, PMMA standard) *M*_n_ = 38.9 kg mol^-1^, *Ɖ*_M_ = 1.16.

*Characterisation of PCL_50_-b-PnOMA_150_*

^1^H NMR (300 MHz, CDCl_3,_ 298 K) δ (ppm) 4.06 (t, *J* = 6.7 Hz, 50H), 3.91 (s, 146H), 3.65 (t, *J* = 6.4 Hz, 2H), 3.21 (q, *J* = 7.5 Hz, 1H), 2.31 (t, *J* = 7.5 Hz, 50H), 1.89 (s, 3H), 1.79 (s, 3H), 1.72 – 1.50 (m, 467H), 1.34 (d, *J* = 24.4 Hz, 1142H), 1.02 (s, 92H), 0.90 (t, *J* = 5.8 Hz, 667H). SEC (0.5% NEt_3_ in CHCl_3_, λ = 309 nm, PMMA standard) *M*_n_ = 25.8 kg mol^-1^, *Ɖ*_M_ = 1.15.

*Sonication of PCL_50_-b-PLMA_150_ cylinders*

Self-nucleated cylinders of PCL_50_-*b*-PLMA_150_ in octane, that had been aged for 9 months post self-assembly, were diluted to 0.5 mg mL^-1^. They were sonicated in a Elmasonic S 15 sonic bath (0 °C) operating at 35 W and a frequency of 50 – 60 Hz for 20 minutes at a time. Aliquots of solution were analysed by TEM imaging as the sonication progressed. Cylinder solution was sonicated for a total of 1 h 40 minutes to provide seed micelles. Seed length was calculated by using ImageJ software to hand count around 100 particles from multiple TEM micrographs. Mean length and standard deviation were calculated by ImageJ software.

*Typical epitaxial growth of unimer to seed solution.*

10 μL of PCL_50_-*b*-PLMA_150_ dissolved in chloroform (10 mg mL^-1^) was added to the dispersion of seed solution (0.01 mg mL^-1^) and aged for 7 days prior to TEM analysis. The unimer-to-seed ratio was altered by changing the concentration of unimer in chloroform added to the seed solution. Theoretical cylinder length is calculated by multiplying the seed length by the seed:unimer mass ratio and adding the initial seed length. For example, the theoretical cylinder length of the example here (PCL_50_-*b*-PLMA_150_ unimer added to seeds of PCL_50_-*b*-PLMA_150_ in a 1:10 ratio: 41 + (41 × 10) = 451 nm. Cylinder length was calculated by using ImageJ software to hand count circa 100 particles from multiple TEM micrographs. Mean length and standard deviation were calculated by ImageJ software.

**Supplementary Figures and Tables**


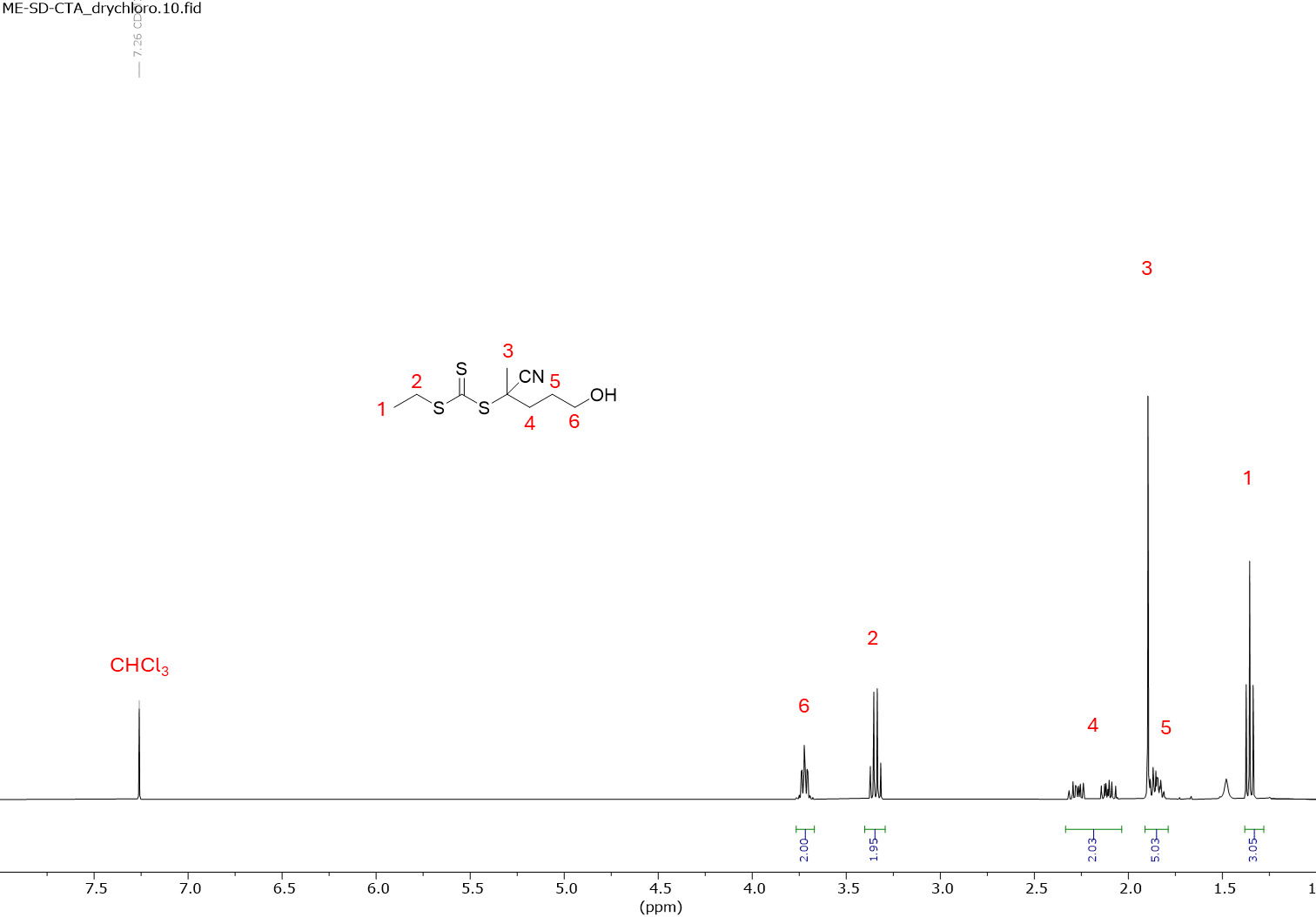


**Figure S1** ^1^H NMR spectrum (CDCl_3_, 400 MHz, 298 K) of 2-cyano-5-hydroxypentan-2-yl ethyl carbonotrithioate (CHPET) dual headed CTA and ROP initiator


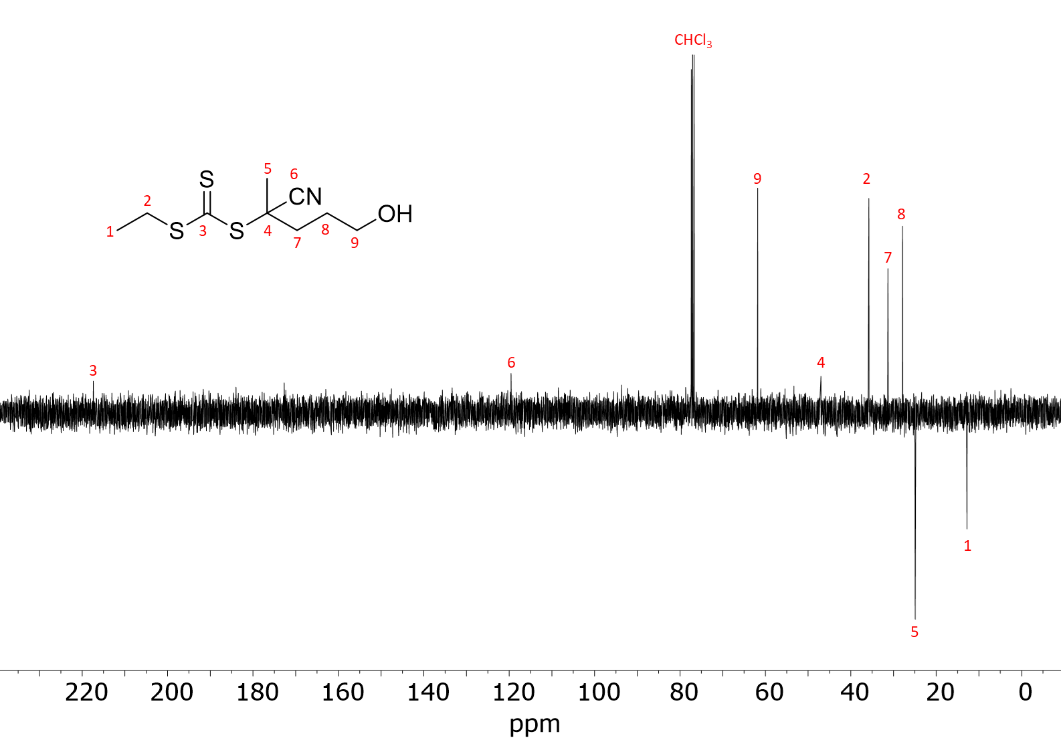


**Figure S2** ^13^C NMR spectrum (CDCl_3_, 101 MHz, 298 K) of 2-cyano-5-hydroxypentan-2-yl ethyl carbonotrithioate (CHPET) dual headed CTA and ROP initiator


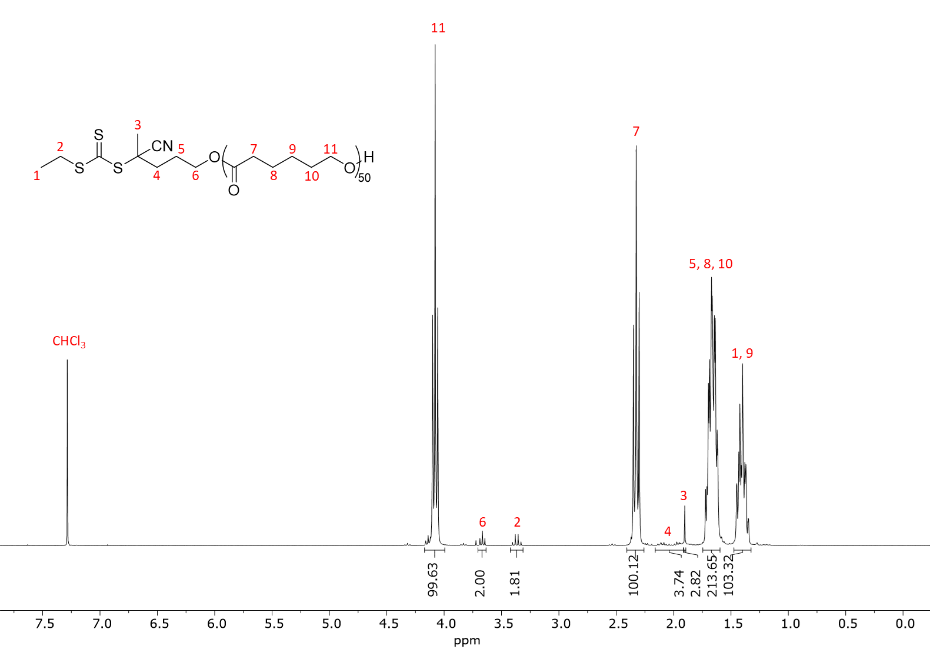


**Figure S3** ^1^H NMR spectrum (CDCl_3_, 300 MHz, 298 K) of PCL_50_


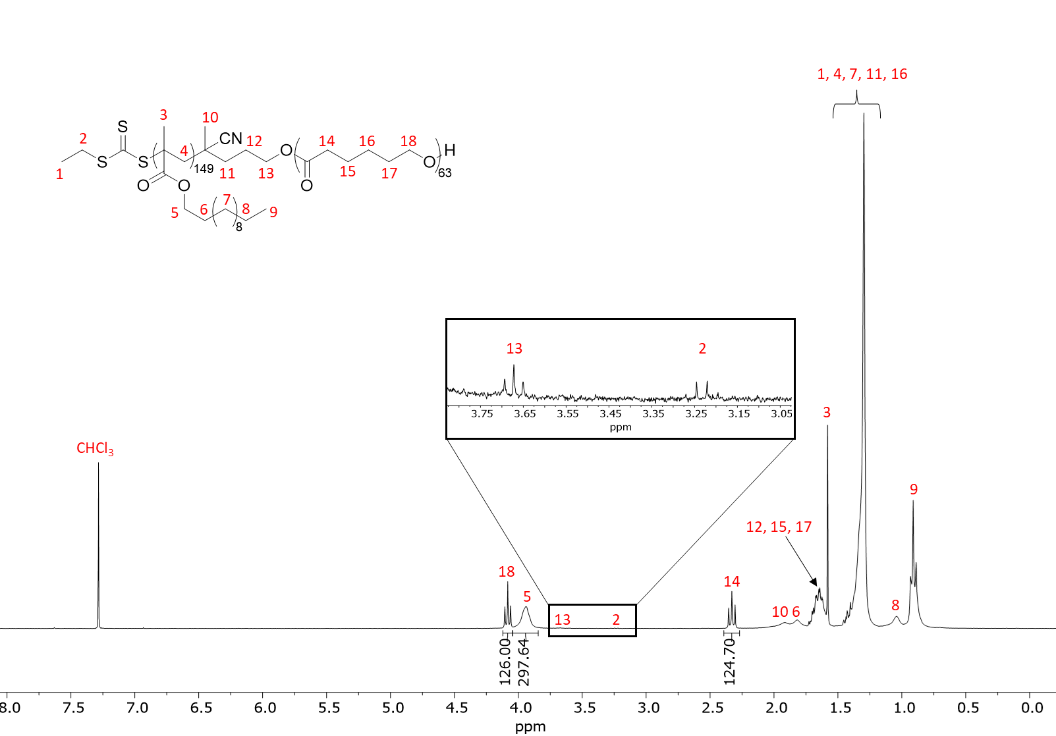


**Figure S4** ^1^H NMR spectrum (CDCl_3_, 300 MHz, 298 K) of PCL_50_-b-PLMA_150_


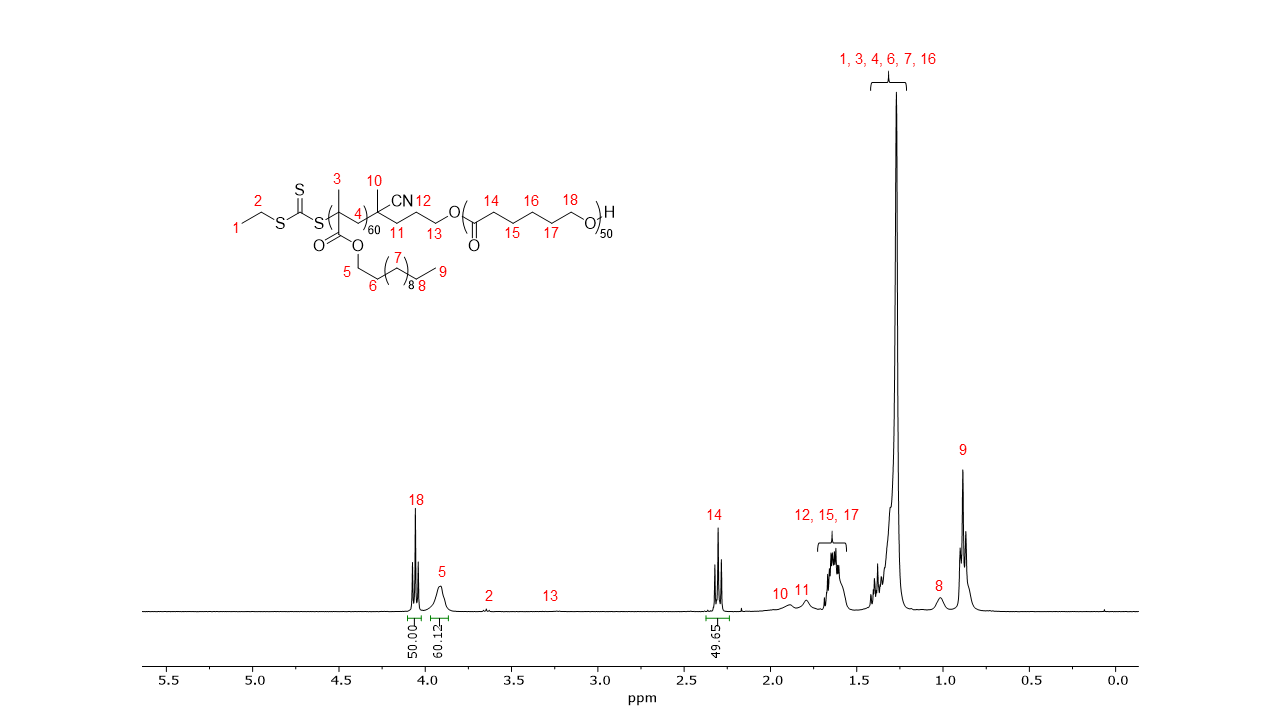


**Figure S5** ^1^H NMR spectrum (CDCl_3_, 300 MHz, 298 K) of PCL_50_-b-PLMA_60_


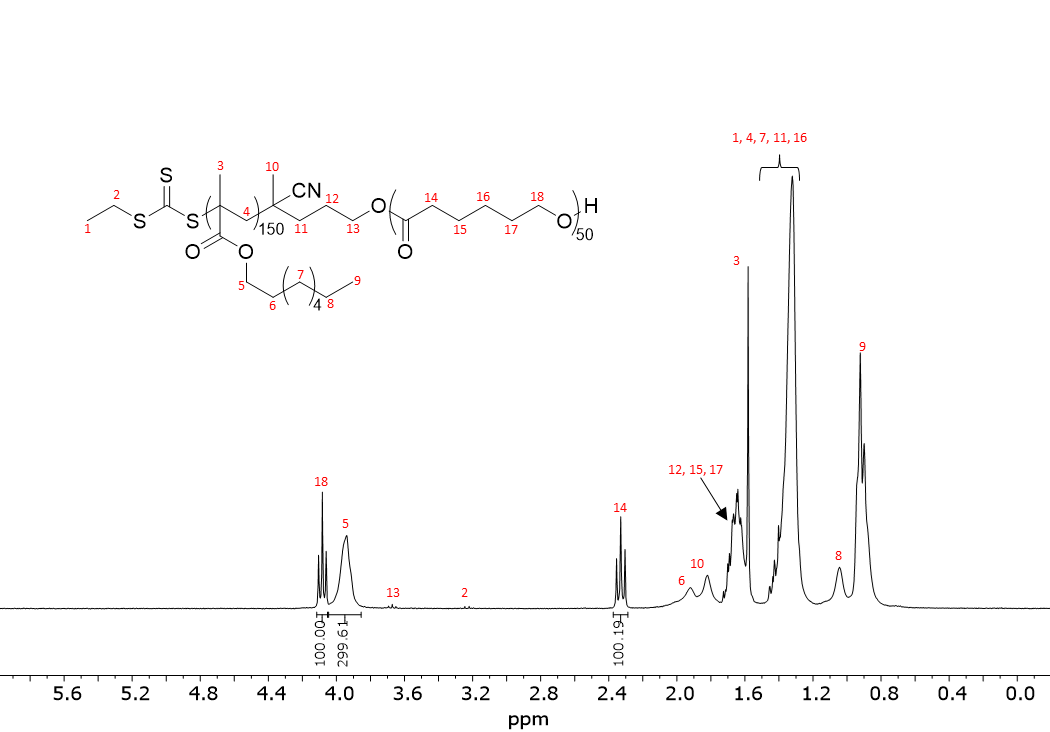


**Figure S6** ^1^H NMR spectrum (CDCl_3_, 300 MHz, 298 K) of PCL_50_-b-PnOMA_146_


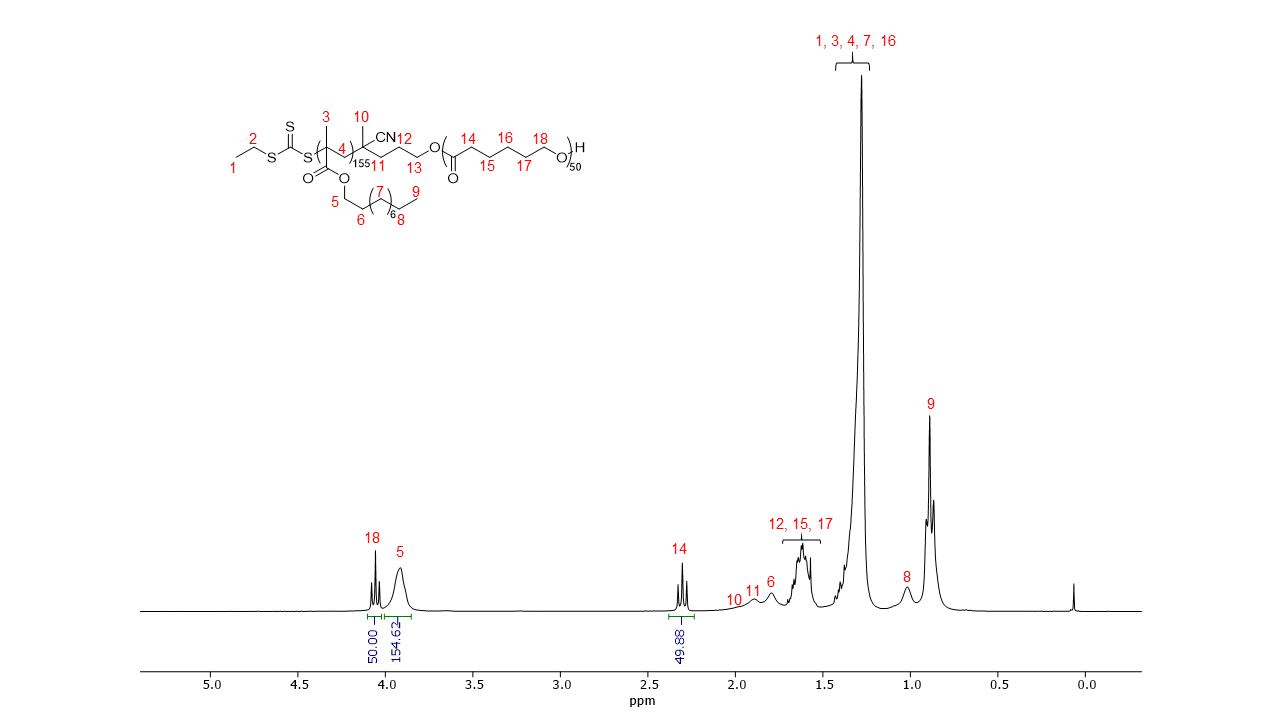


**Figure S7** ^1^H NMR spectrum (CDCl_3_, 300 MHz, 298 K) of PCL_50_-b-PnDMA_155_

**
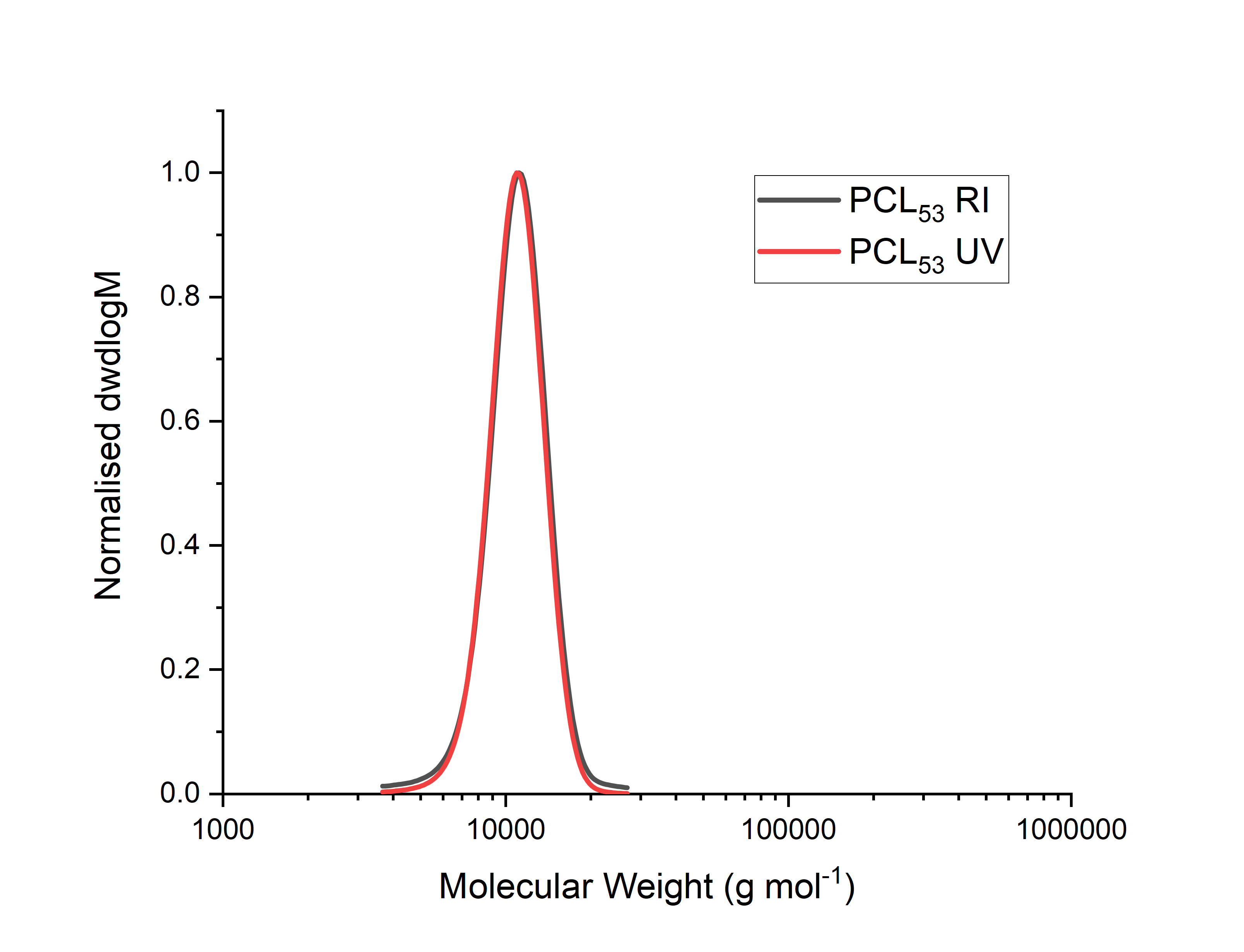
**

**Figure S8** Overlaid RI and UV (λ = 309 nm) SEC chromatograms of PCL_50_ using CHCl_3_ with 0.5% NEt_3_ as an eluent with poly(methyl methacrylate) (PMMA) as a standard


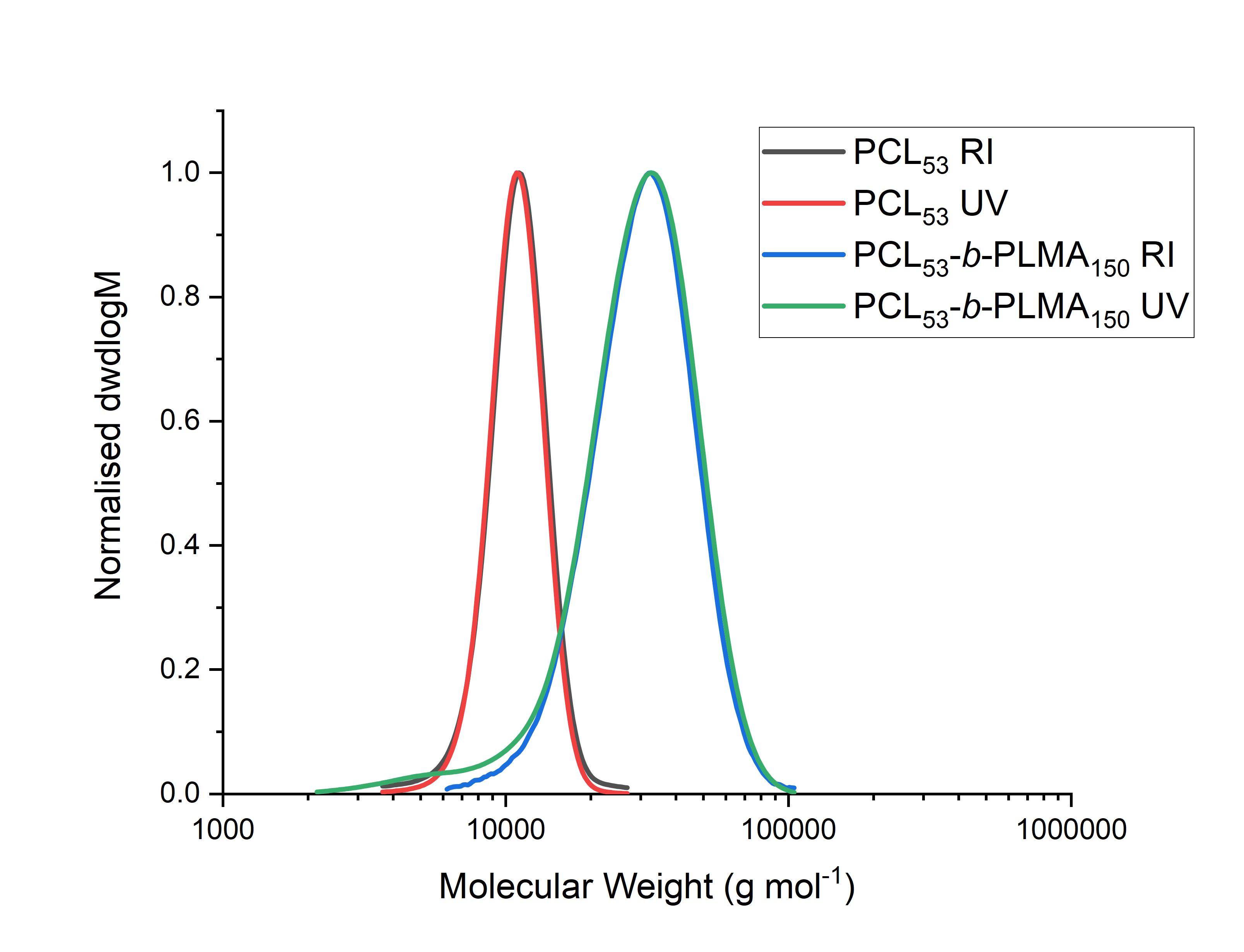


**Figure S9** Overlaid RI and UV (λ = 309) SEC chromatograms of PCL_50_-b-PLMA_150_ using CHCl_3_ with 0.5% NEt_3_ as an eluent with PMMA as a standard


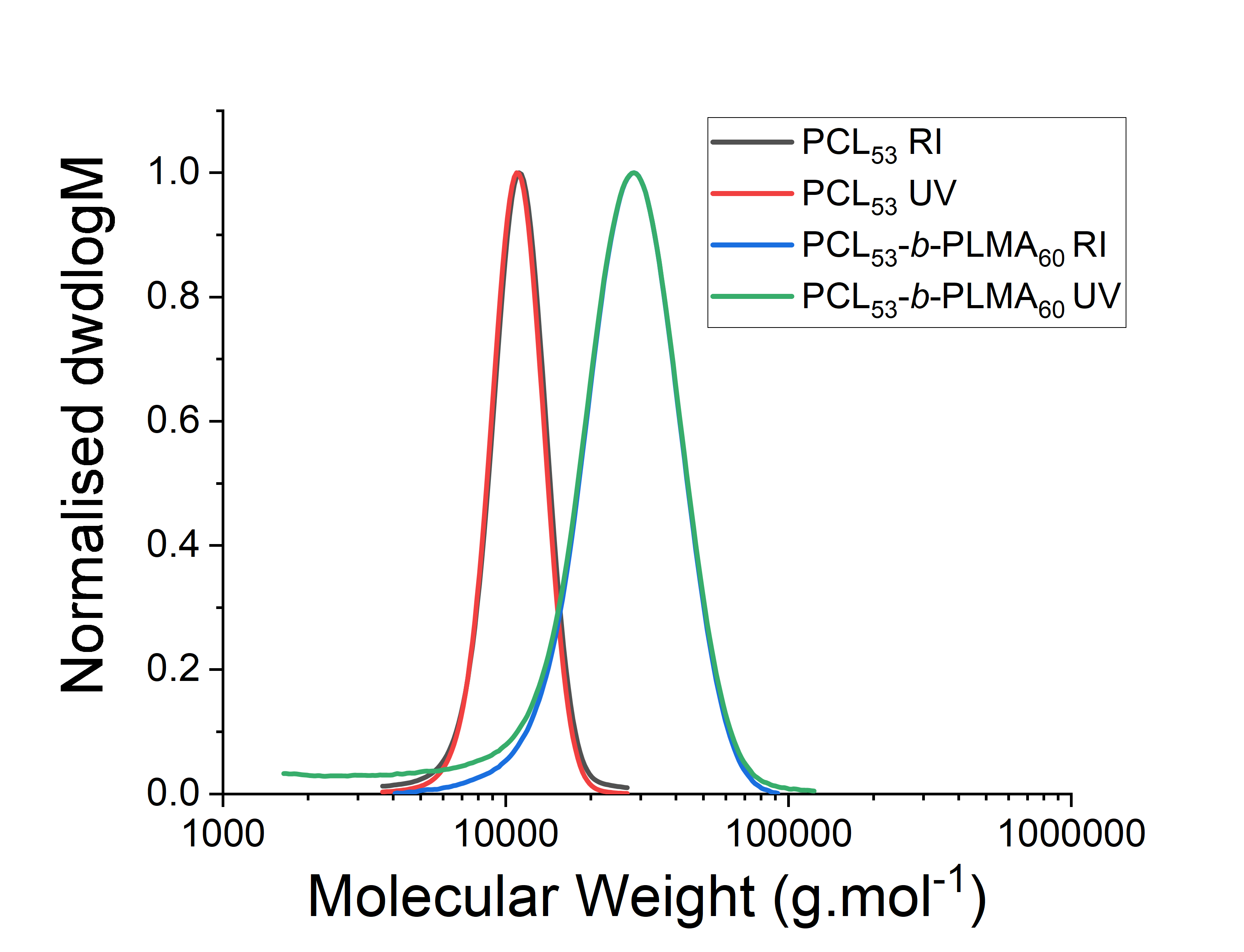


**Figure S10** Overlaid RI and UV (λ = 309) SEC chromatograms of PCL_50_-b-PLMA_60_ using CHCl_3_ with 0.5% NEt_3_ as an eluent with PMMA as a standard


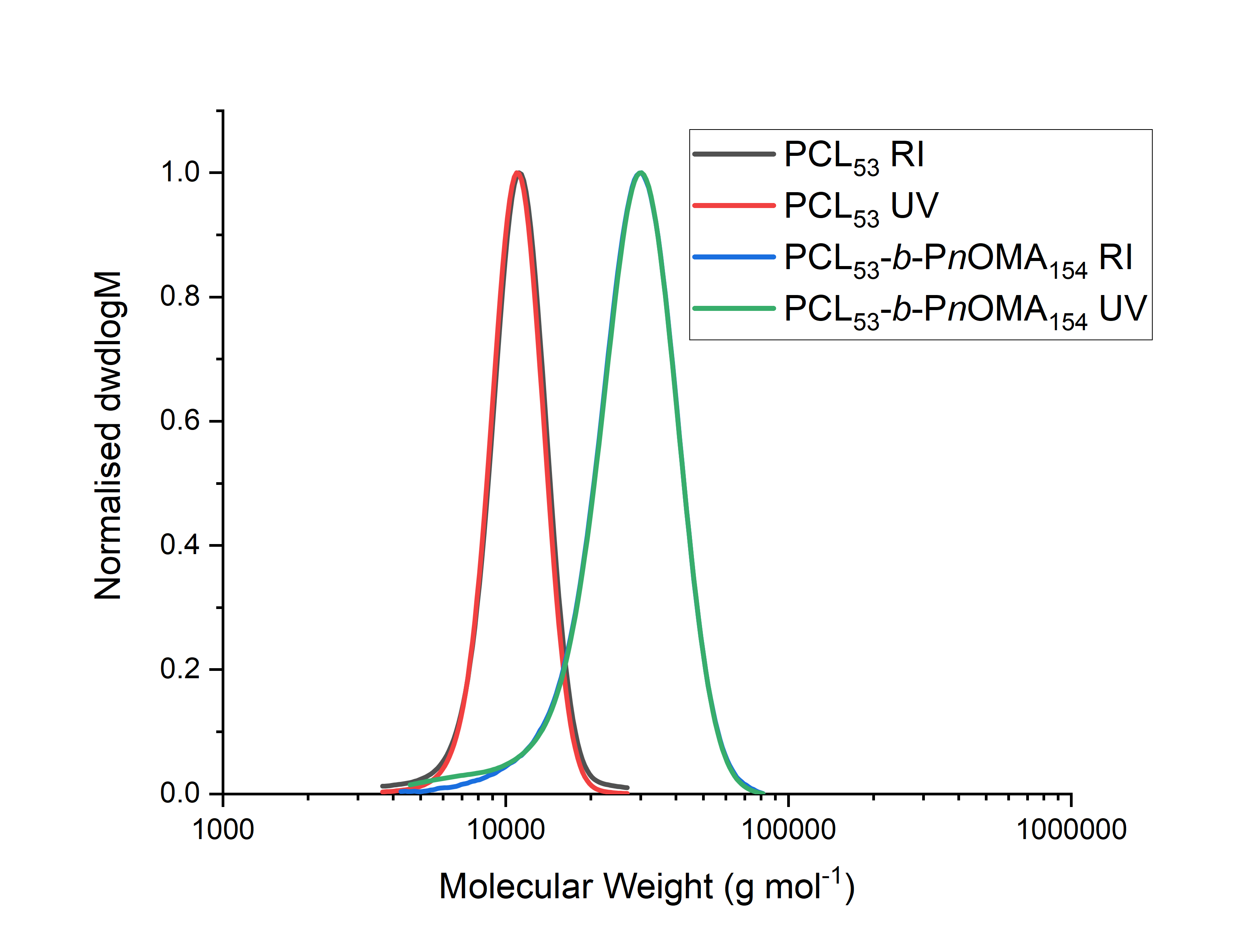


**Figure S11** Overlaid RI and UV (λ = 309) SEC chromatograms of PCL_50_-b-PnOMA_146_ using CHCl_3_ with 0.5% NEt_3_ as an eluent with PMMA as a standard

**Figure S12** Overlaid RI and UV (λ = 309) SEC chromatograms of PCL_50_-b-PnDMA_155_ using CHCl_3_ with 0.5% NEt_3_ as an eluent with PMMA as a standard

Endo

**Figure S13** Differential scanning calorimetry thermogram of PCL_50_-b-PLMA_150_ carried out at 10 °C min^-1^

Endo

**Figure S14** Differential scanning calorimetry thermogram of PCL_50_-b-PLMA_60_ carried out at 10 °C min^-1^

Endo

**Figure S15** Differential scanning calorimetry thermogram of PCL_50_-*b*-P*n*OMA_150_ carried out at 10 °C min^-1^


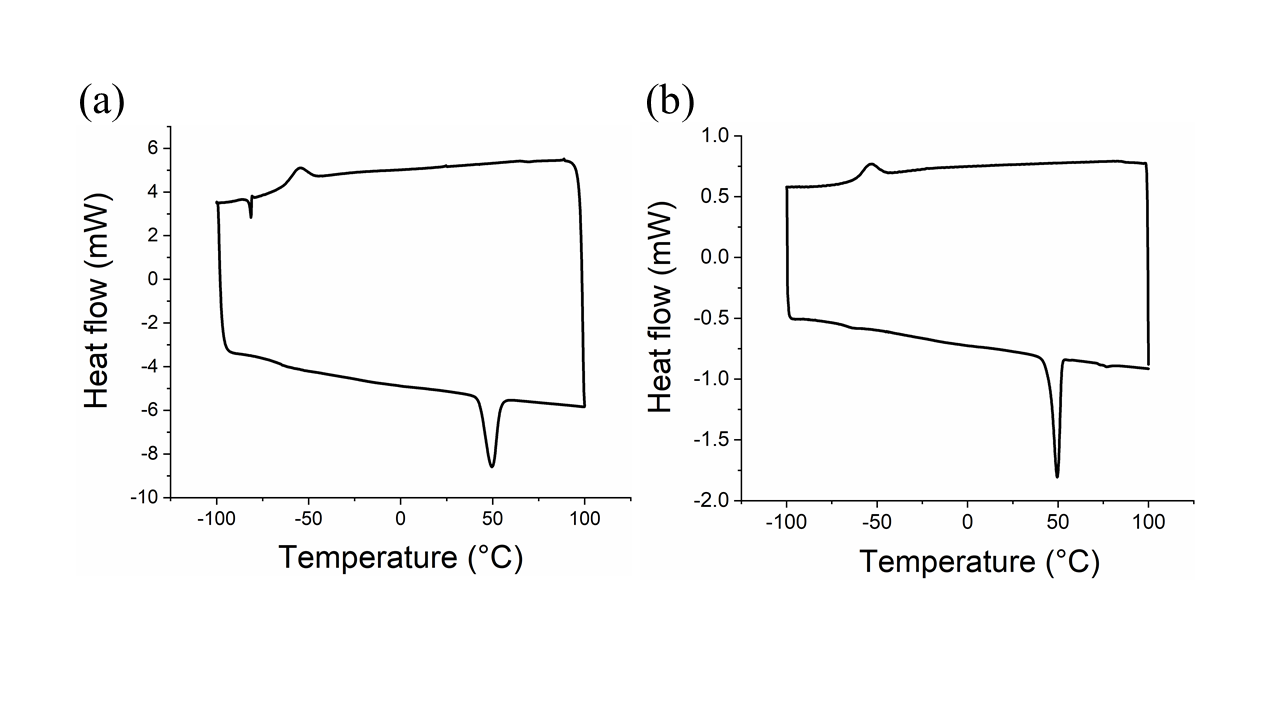


Endo

**Figure S16** Differential scanning calorimetry thermogram of PCL_50_-*b*-P*n*DMA_155_ carried out at 10 °C min^-1^

**Scheme S1** Overview of synthesis of RAFT BCPs from polymerisation of ɛ-caprolactone where n= degree of polymerisation and m= alkyl chain length

**Table S1** Characterization of PCL_50_-b-PLMA_150_, PCL_50_-b-PLMA_60_, PCL_50_-b-PnDMA_155_, and PCL_50_-b-PnOMA_146_

| **Polymer** | ***M*_n_^(SEC)^** / kg mol^-1^ | ***M*_n_^(NMR)^** / kg mol^-1^ | ***Ɖ*_M_** | ***T*_m_**  **(°C)** | ***T*_c_**  **(°C)** | ***T*_g_**  **(°C)** |
| --- | --- | --- | --- | --- | --- | --- |
| PCL_50_ | 6.0 | 10.5 | 1.07 | - | - | - |
| PCL_50_-b-PLMA_150_ | 44.1 | 30.4 | 1.18 | 48.1 | -44.0 | -39.1 |
| PCL_50_-b-PLMA_60_ | 21.2 | 24.9 | 1.18 | 49.2 | -41.0 | -35.4 |
| PCL_50_-b-PnOMA_146_ | 34.9 | 25.8 | 1.15 | 49.5 | -52.9 | -65.8 |
| PCL_50_-b-PnDMA_155_ | 41.1 | 38.9 | 1.16 | 49.5 | -54.6 | -64.9 |

^a^Observed molecular weight as calculated by ^1^H NMR spectroscopic end group analysis. ^b^Molecular weight as calculated by SEC analysis using CHCl_3_ as an eluent with PMMA standards.

**Figure S17** DLS and correlogram of PCL_50_-*b*-PLMA_60_ after two weeks of aging

**Figure S18** DLS and correlogram of PCL_50_-*b*-PLMA_150_ after two weeks of aging

**Figure S19** DLS and correlogram of PCL_50_-*b*-P*n*OMA_60_ after two weeks of aging

**Figure S20** DLS and correlogram of PCL_50_-*b*-P*nD*MA_60_ after two weeks of aging

Endo

Endo

**Figure S21** nano-DSC of PCL-*b*-P*n*DMA_155_ in octane at 5 mg mL^-1^ after two days of aging carried out at 1 °C min^-1^

Endo

**Figure S22** nano-DSC of PCL-*b*-P*n*OMA_150_ in octane at 5 mg mL^-1^ after two weeks of aging carried out at 1 °C min^-1^

Endo

**Figure S23** nano-DSC of PCL-*b*-PLMA_60_ in octane at 5 mg mL^-1^ after two weeks of aging carried out at 1 °C min^-1^

Endo

**Figure S24** nano-DSC of PCL-*b*-PLMA_150_ in octane at 5 mg mL^-1^ after two days of aging carried out at 1 °C min^-1^


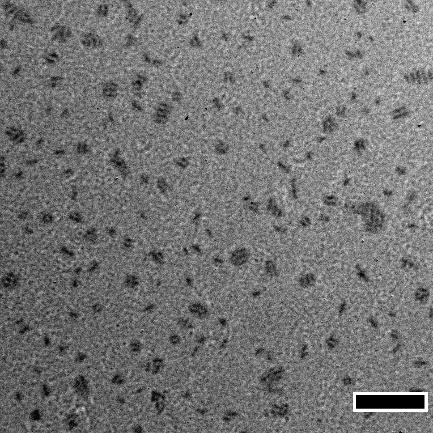


**Figure S25** TEM micrograph of cylindrical micelles of PCL_50_-b-PLMA_150_ after sonication for 1 h 40 minutes (Left) and Histogram showing the length distribution of cylindrical micelles of PCL_50_-b-PLMA_150_ after sonication for 1 h 40 minutes by counting ~100 particles (right). Scale bars = 500 nm


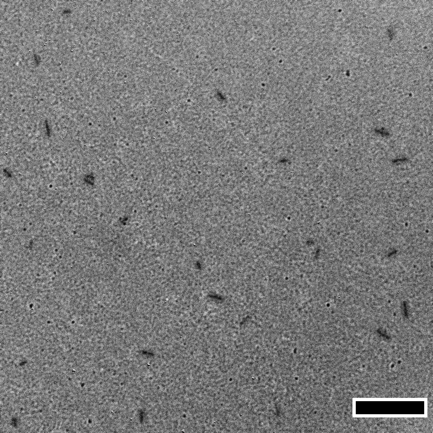

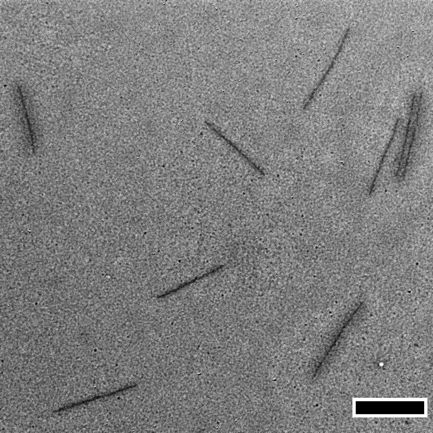

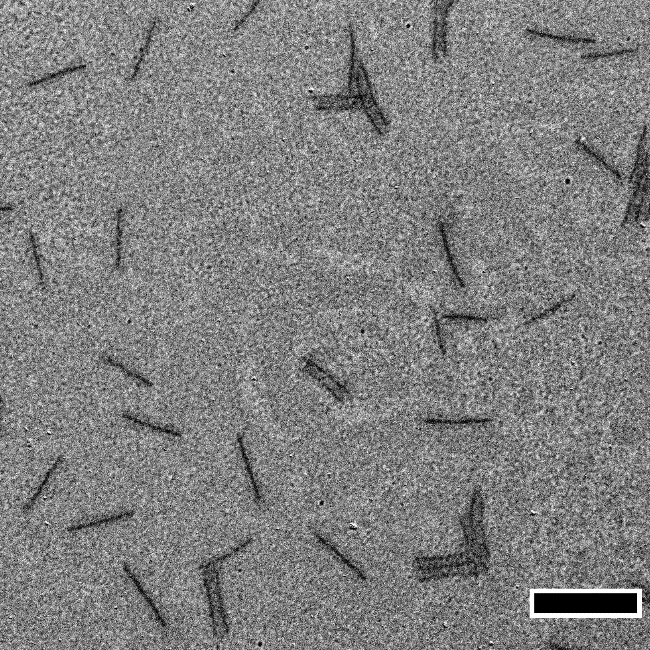


**Figure S26** TEM micrographs of living-CDSA solutions of PCL_50_-b-PLMA_150_ after 56 days of aging at room temperature with m_unimer_/m_seed_ ratios 1, 10, and 25 (Left to right). Scale bars = 500 nm

**Figure S27** Plot showing the linear relationship between different *m*_unimer_/*m*_seed_ ratios with narrow length dispersity (error bars represent standard deviation), compared to the theoretically expected cylinder length of PCL_50_-*b*-PLMA_150_

**Table S2** Average cylinder length of PCL_50_-b-PLMA_150_ after 7, 25, 35, and 56 days of aging at room temperature

| ***m*_unimer_*/***  ***m*_seed_** | **Theoretical *L*_ave_ (nm)** | ***L*_ave_ after 7 days (nm)** | ***L*_ave_ after 25 days (nm)** | ***L*_ave_ after 35 days (nm)** | ***L*_ave_ after 56 days (nm)** |
| --- | --- | --- | --- | --- | --- |
| Seeds | 41 ± 11 | 41 ± 11 | 41 ± 11 | 41 ± 11 | 41 ± 11 |
| 1 | 82 | 61 ± 10 | 84 ± 17 | 76 ± 15 | 70 ± 16 |
| 10 | 451 | 220 ± 21 | 280 ± 33 | 278 ± 34 | 303 ± 42 |
| 25 | 1,064 | 381 ± 44 | 534 ± 47 | 563 ± 73 | 613 ± 57 |


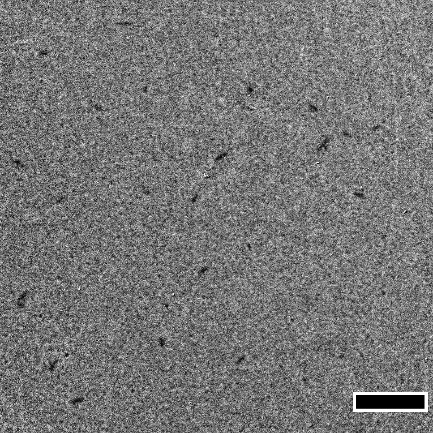

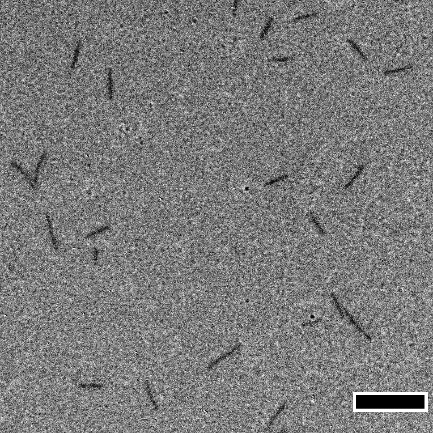

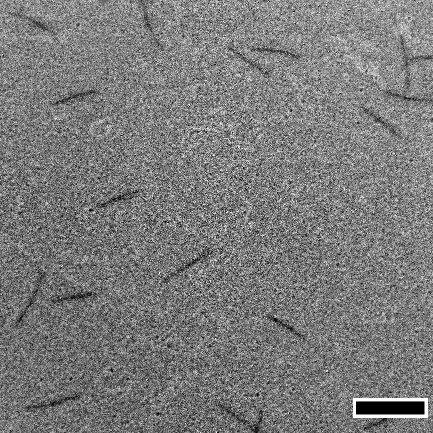


**Figure S28** TEM micrographs of living-CDSA solutions of PCL_50_-*b*-PLMA_150_ after 35 days of aging at 4 °C with *m*_unimer_/*m*_seed_ ratios 1, 10, 25 (Left to right). Scale bar = 500 nm

**Figure S29** Plot showing the linear relationship between different *m*_unimer_/*m*_seed_ ratios with narrow length dispersity of cylindrical nanoparticles of PCL_50_-*b-*PLMA_150_after 35 days aged at room temperature and at 4 °C (error bars represent standard deviation), compared to the theoretically expected length.

**Table S3** Average cylinder length of PCL_50_-b-PLMA_150_ after 7, 14, and 35 days of aging at 4 °C

| ***m*_unimer_*/***  ***m*_seed_** | **Theoretical *L*_ave_ (nm)** | ***L*_ave_ after 7 days at 4 °C (nm)** | ***L*_ave_ after 14 days at 4 °C (nm)** | ***L*_ave_ after 35 days at 4 °C (nm)** |
| --- | --- | --- | --- | --- |
| Seeds | 41 ± 11 | 41 ± 11 | 41 ± 11 | 41 ± 11 |
| 1 | 82 | 56 ± 13 | 58 ± 14 | 53 ± 13 |
| 10 | 451 | 140 ± 27 | 144 ± 37 | 184 ± 44 |
| 25 | 1,064 | 219 ± 41 | 247 ± 62 | 332 ± 62 |

**Figure S30** Average cylinder lengths of PCL_50_-*b*-PLMA_150_ from living growth, *m*_unimer_*/m*_seed_ = 10 with increasing volume percentage of heptane co-solvent after 7 days. Error bars represent standard deviation

**Table S4** Average cylinder length of PCL_50_-*b*-PLMA_150_ with increasing percentage volume of heptane after 7 days

| **Percentage volume heptane (%)** | **L_ave_ after 7 days at room temperature (nm)** |
| --- | --- |
| 0 | 217 ± 34 |
| 1 | 237 ± 40 |
| 3 | 215 ± 33 |
| 7 | 215 ± 36 |
| 10 | 216 ± 34 |
| 15 | 206 ± 42 |
| 20 | 269 ± 42 |
| 30 | 272 ± 40 |
| 40 | 285 ± 51 |
| 50 | 267 ± 43 |


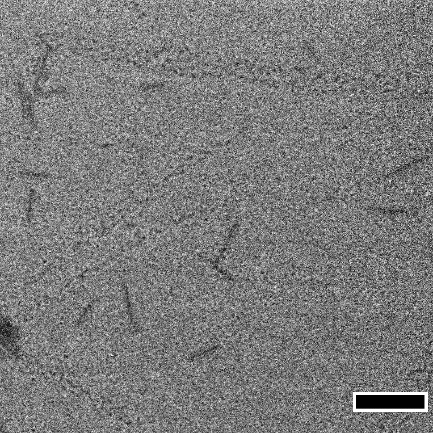

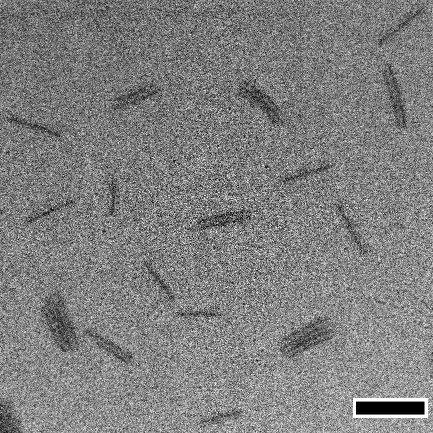


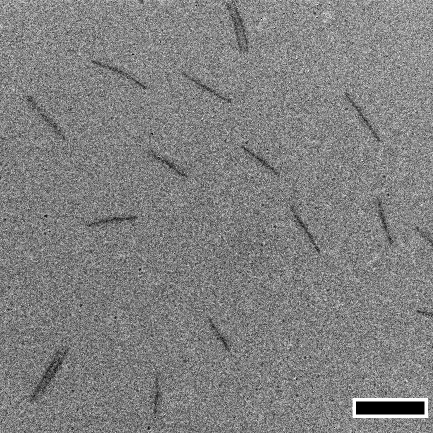

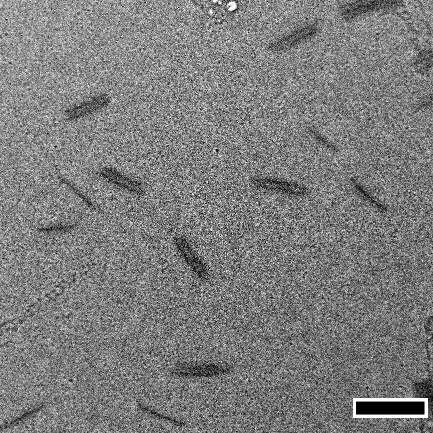


**Figure S31** TEM micrographs of living growth of PCL_50_-b-PLMA_150_ in octane with 40 %vol. heptane after 7 days, 19 days (top row), 35 days, and 49 days (bottom row). Scale bars = 500 nm

**Figure S32** Comparison of L_ave_ over time for the living growth of PCL_50_-b-PLMA_150_ in octane and octane/heptane 60/40 v/v %

**Table S5** Comparisons of L_ave_ after aging in octane and octane/heptane 60/40 v/v %

| **Octane** | | **Octane/heptane 60/40 v/v %** | |
| --- | --- | --- | --- |
| **Days aged** | **L_ave_ (nm)** | **Days aged** | **L_ave_ (nm)** |
| 7 | 220 ± 21 | 7 | 285 ± 51 |
| 25 | 280 ± 33 | 19 | 345 ± 44 |
| 35 | 278 ± 34 | 35 | 374 ± 48 |
| 56 | 303 ± 42 | 49 | 376 ± 46 |

**Table S6** Cylinder lengths from direct addition of dissolved unimer of PCL_50_-b-PLMA_60_ to seed nanoparticle solutions after 7 days

| ***m*_unimer_/**  ***m*_seed_** | **Theoretical *L*_ave_ (nm)** | ***L*_ave_ after 7 days (nm)** |
| --- | --- | --- |
| Seeds | 41 ± 11 | 41 ± 11 |
| 1 | 123 | 109 ± 25 |
| 3 | 286 | 309 ± 42 |
| 7 | 612 | 747 ± 65 |
| 10 | 857 | 982 ± 99 |
| 25 | 2,091 | 708 ± 462 |


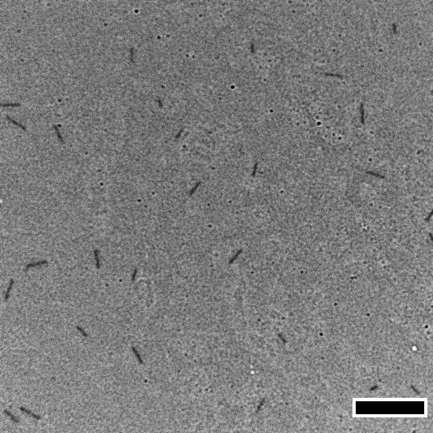

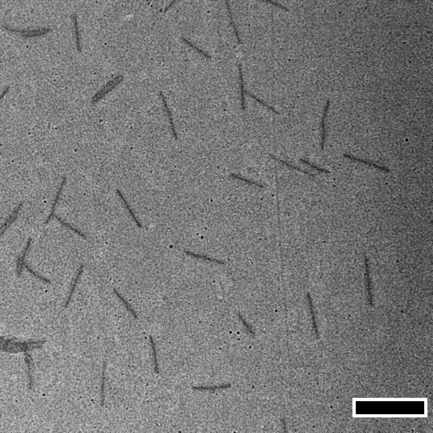


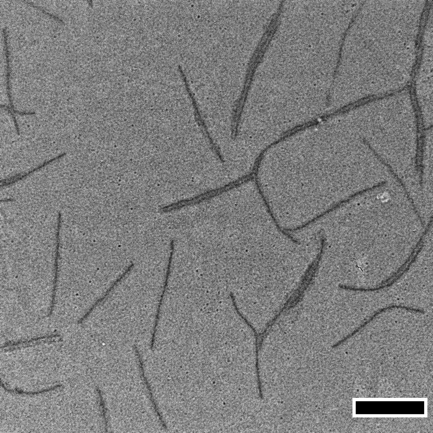

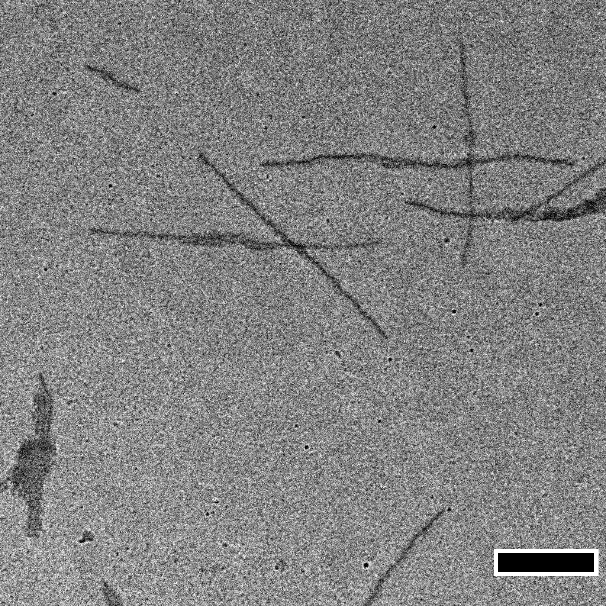


**Figure S33** TEM micrographs of living growth solutions of PCL_50_-*b*-PLMA_60_ unimer added to seeds of PCL_50_-*b*-PLMA_150_ in octane with *m*_unimer_/*m*_seed_ ratio 1, 3 (Top row), and 7, 25 (Bottom row). Scale bar = 500 nm

**Figure S34** Plot showing the relationship between different m_unimer_/m_seed_ ratios of PCL_50_-b-PLMA_60_, with narrow length dispersity of cylindrical nanoparticles up to a ratio of 10 (error bars represent standard deviation), compared to the theoretically expected cylinder length.


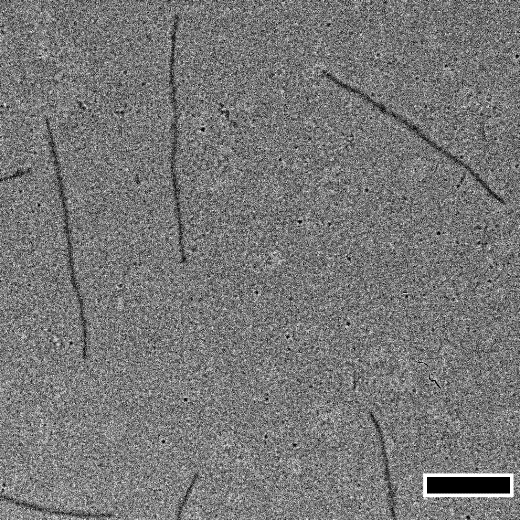

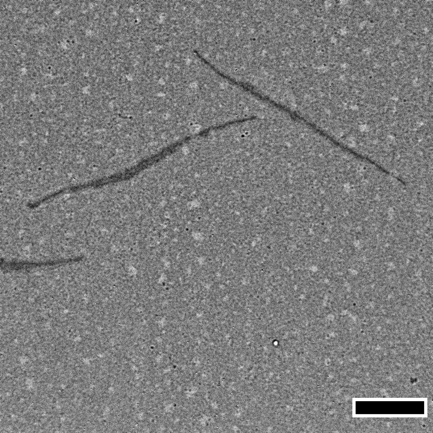


**Figure S35** TEM micrographs of living growth solution of PCL_50_-*b*-PLMA_60_, by sequential addition of unimer to achieve final *m*_unimer_/*m*_seed_ ratio of 15, and 20 (Left to right), to seeds of PCL_50_-b-PLMA_150_ in octane. Scale bars = 500 nm.

**Figure S36** Histogram showing cylinder lengths measured in living growth solutions with *m*_unimer_*/m*_seed_ = 15 and 25 after sequential additions of unimer.

**Table S7** Table of cylinder length from three and five sequential additions of dissolved unimer to seed nanoparticle solution.

| ***m_unimer_/m_seed_*** | **Theoretical *L*_ave_ (nm)** | **Measured *L*_ave_ (nm)** |
| --- | --- | --- |
| 15 | 1,265 | 1,455 ± 123 |
| 25 | 2,091 | 1,561 ± 335 |


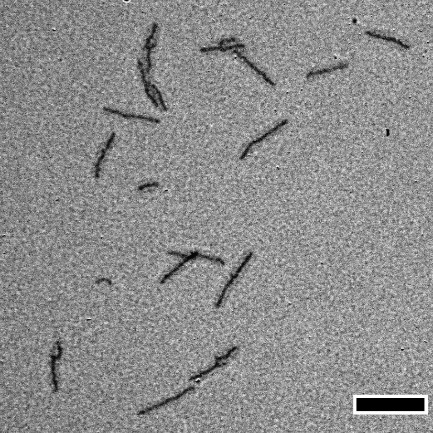


**Figure S37** TEM micrograph of living growth solution of PCL_50_-b-PnOMA_146_ unimer added to seeds of PCL_50_-b-PLMA_150_ in octane with m_unimer_/m_seed_ ratio of 10 after 7 days. Scale bar = 500 nm (left). Histogram showing the distribution of cylinder lengths (right).


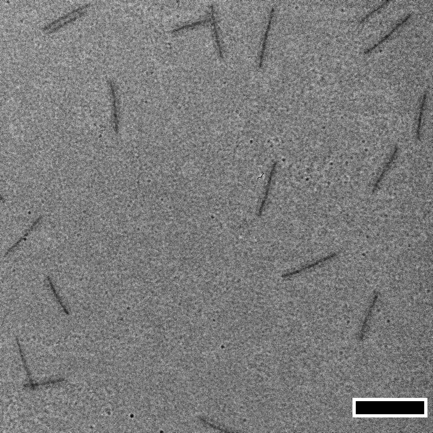

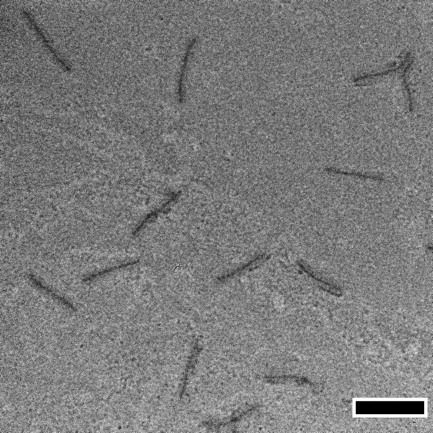


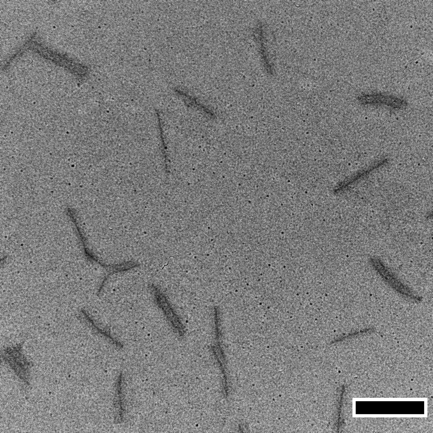

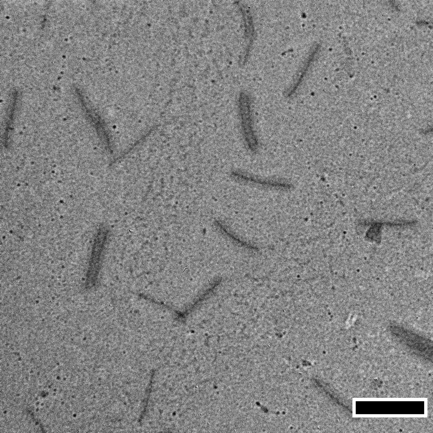


**Figure S38** TEM micrographs of 1D epitaxial growth of PCL_50_-*b*-P*n*DMA_155_ from seeds of PCL_50_-*b*-PLMA_150_ in a *m*_unimer_/*m*_seed_ ratio of 10 after 7, 14 (Top row), and 33, 67 days (Bottom row). Scale bars = 500 nm

**Table S8** Cylinder length of PCL_50_-b-PnDMA_155_ grown from seeds of PCL_50_-b-PLMA_150_ over time

| **Days aged** | **Theoretical *L*_ave_ (nm)** | ***L*_ave_ _of_ ~100 particles (nm)** |
| --- | --- | --- |
| Seeds | 41 ± 11 | 41 ± 11 |
| 7 | 478 | 348 ± 45 |
| 14 | 478 | 383 ± 46 |
| 33 | 478 | 435 ± 42 |
| 67 | 478 | 458 ± 42 |

**Table S9** Cylinder lengths of PCL_50_-*b*-P*n*DMA_155_ grown from seeds of PCL_50_-*b*-PLMA_150_ after 67 days for *m*_unime_*_r_/m*_seed_ ratios 1, 10, and 25

| ***m*_unimer_*/***  ***m*_seed_** | **Theoretical *L*_ave_ (nm)** | ***L*_ave_ after 67 days (nm)** |
| --- | --- | --- |
| Seeds | 41 ± 11 | 41 ± 11 |
| 1 | 85 | 67 ± 14 |
| 10 | 478 | 458 ± 42 |
| 25 | 1134 | 1014 ± 137 |


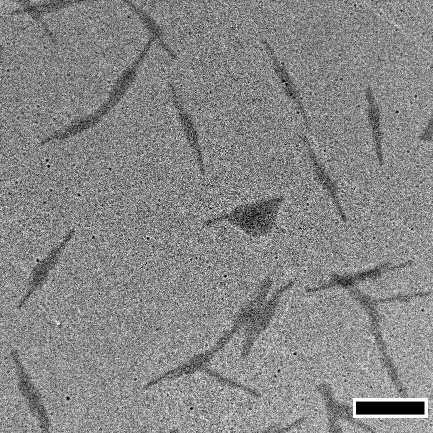

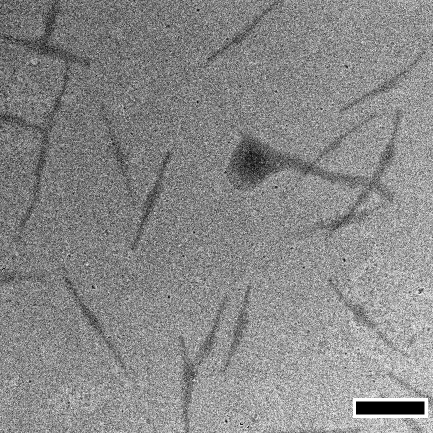


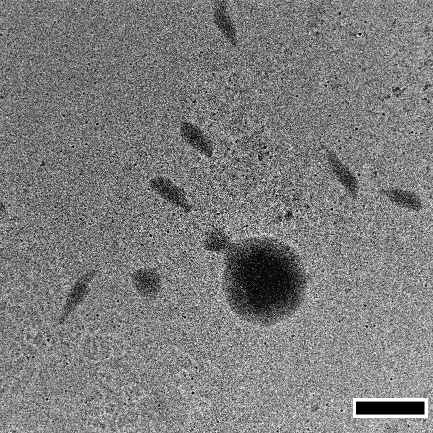

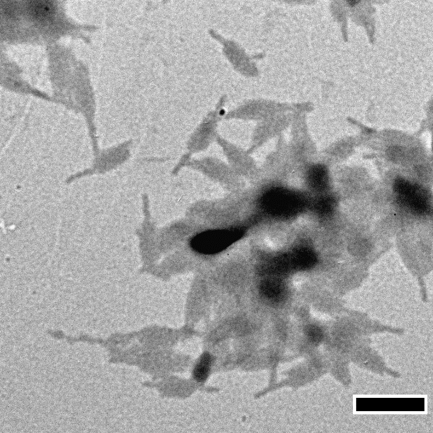


**Figure S39** TEM micrographs of living growth solutions of PCL_50_-*b*-PLMA_60_ unimer co-crystallised with PCL_50_ homopolymer onto seeds of PCL_50_-*b*-PLMA_150_ where *m*_unimer_/*m*_seeds_ = 10, with *m*_PCL50_/*m*_unimer_ ratios 0.25, 0.5 (Top row), and 1, 2 (Bottom row). Scale bars = 500 nm

**Figure S40** Measured platelet lengths (top) and measured platelet widths (bottom) of PCL_50_-*b*-PLMA_150_. Large PCL platelet outliers have been removed to highlight platelets assembled by controlled epitaxial growth

**Table S10** Length and width values of platelets of PCL_50_-*b*-PLMA_150_ co-crystallised with PCL_50_ when *m*_PCL50_/*m*_unimer_ = 10 and *m*_PCL50_*/m*_unimer_ = 1 after 7, 35 and 135 days

|  | **Length (nm)** | | | **Width (nm)** | | | **Length/width** | |
| --- | --- | --- | --- | --- | --- | --- | --- | --- |
| **Time (days)** | ***L_n_*** | ***L_w_*** | ***L_w_/L_n_*** | ***W_n_*** | ***W_w_*** | ***W_w_/W_n_*** | ***L_n_/W_n_*** | ***L_w_/W_w_*** |
| 7 | 166 | 168 | 1.01 | 91 | 92 | 1.02 | 1.84 | 1.83 |
| 35 | 205 | 212 | 1.03 | 112 | 123 | 1.10 | 1.84 | 1.72 |
| 135 | 217 | 350 | 1.61 | 124 | 269 | 2.17 | 1.75 | 1.30 |

**Table S11** Length and width values of platelets of PCL_50_-b-PLMA_150_ co-crystallised with PCL_50_ where m_PCL50_/m_unimer_ = 1 and m_unimer_/m_seeds_ ratios = 1, 10, 25, 40, and 60 after 135 days

|  | **Length (nm)** | | | **Width (nm)** | | | **Length/width** | |
| --- | --- | --- | --- | --- | --- | --- | --- | --- |
| ***m*_unimer_*/***  ***m*_seeds_** | ***L_n_*** | ***L_w_*** | ***L_w_/L_n_*** | ***W_n_*** | ***W_w_*** | ***W_w_/W_n_*** | ***L_n_/W_n_*** | ***L_w_/W_w_*** |
| 1 | 129 | 135 | 1.05 | 53 | 59 | 1.11 | 2.41 | 2.27 |
| 10 | 217 | 350 | 1.61 | 124 | 269 | 2.17 | 1.75 | 1.30 |
| 25 | 255 | 272 | 1.07 | 139 | 157 | 1.13 | 1.84 | 1.74 |
| 40 | 291 | 295 | 1.01 | 174 | 175 | 1.01 | 1.67 | 1.69 |
| 60 | 298 | 302 | 1.01 | 187 | 191 | 1.02 | 1.59 | 1.58 |


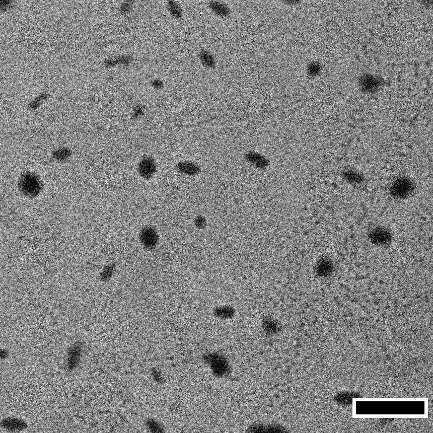

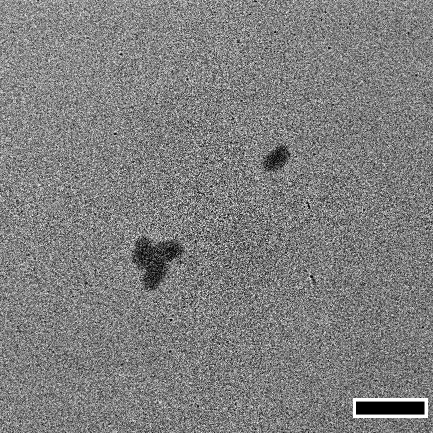

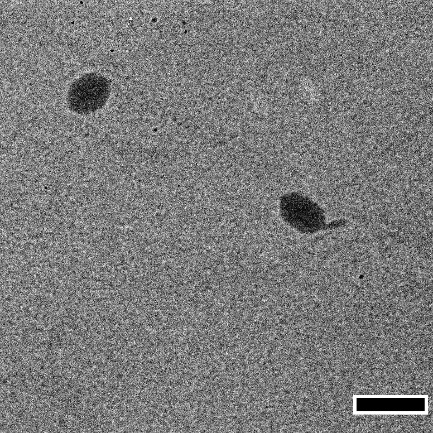

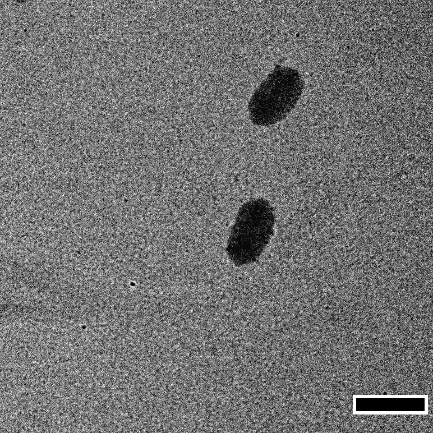

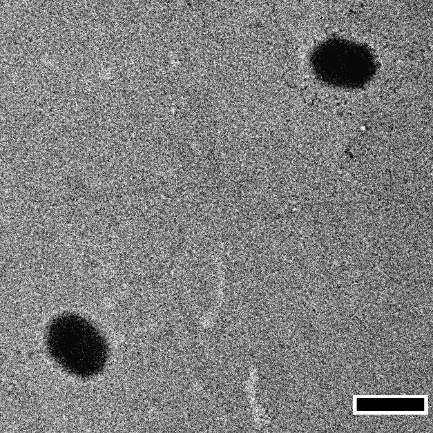

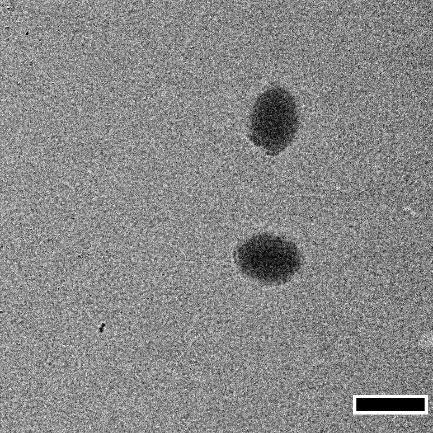


**Figure S41** TEM micrographs of platelet nanoparticles of PCL_50_ and PCL_50_-*b*-PnDMA_155_ co-crystallised from seeds of PCL_50_-*b*-PLMA_150_ in *m*_unimer_/*m*_seed_ ratios 1, 10, 25 (top) and 40, 60, 100 (bottom). Scale bar = 500 nm


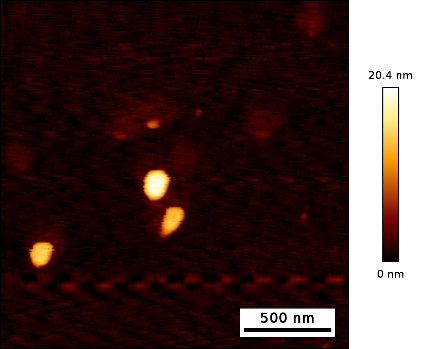

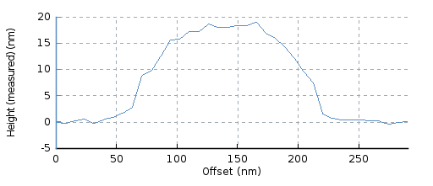


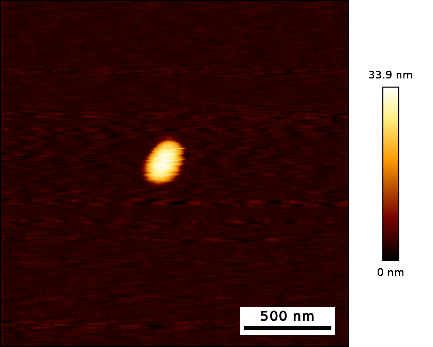

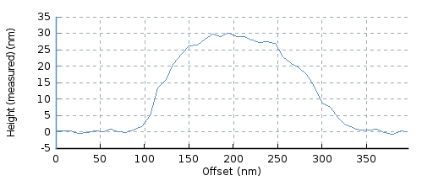


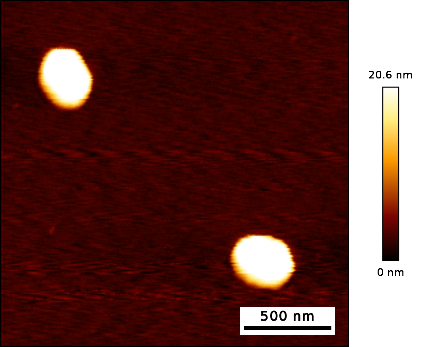

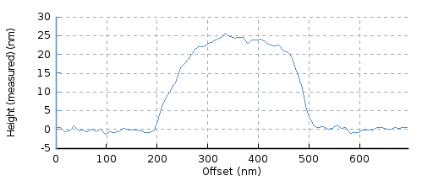


**Figure S42** AFM micrographs with cross-section height measurements of platelet nanoparticles of PCL_50_ and PCL_50_-*b*-P*n*DMA_155_ co-crystallised from seeds of PCL_50_-*b*-PLMA_150_ of *m*_unimer_/*m*_seed_ ratios 1, 10 and 25 (top to bottom)


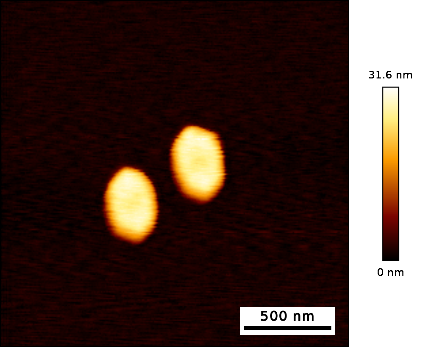

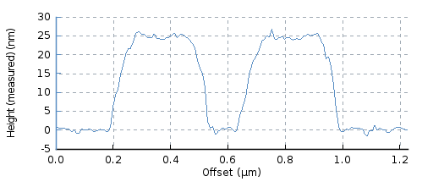


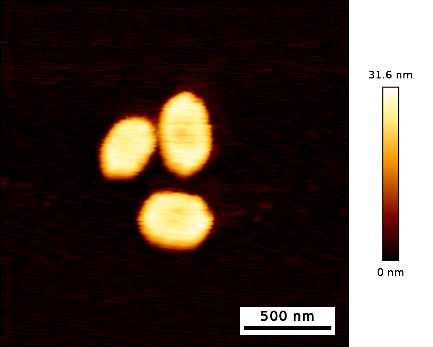

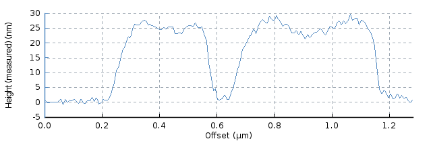


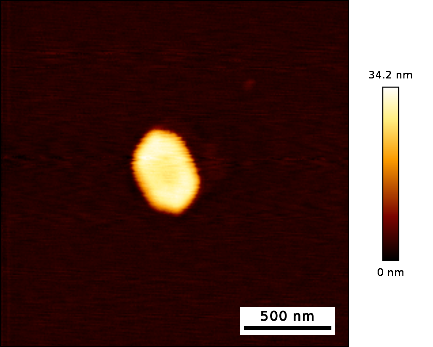

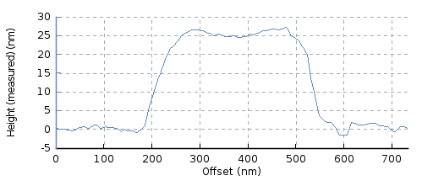


**Figure S43** AFM micrographs with cross-section height measurements of platelet nanoparticles of PCL_50_ and PCL_50_-*b*-P*n*DMA_155_ co-crystallised from seeds of PCL_50_-*b*-PLMA_150_ in *m*_unimer_/*m*_seed_ ratios 40, 60, and 100 (top to bottom)

**Figure S44** Measured platelet lengths (top) and measured platelet widths (bottom) of each *m*_unimer_/*m*_seed_ ratio by measuring ~100 particles of PCL_50_-*b*-P*n*DMA_155_

**Figure S45** Length/Width ratios of each *m*_unimer_/*m*_seed_ ratio of PCL_50_-*b*-P*n*DMA_155_ plates

**Table S12** Measured length and width values of platelets of PCL_50_-b-PnDMA_155_ co-crystallised with PCL_50_ where m_PCL50_/m_unimer_ = 1 and m_unimer_/m_seed_ = 1, 10, 25, 40, 60, and 100, after 60 days

|  | **Length (nm)** | | | **Width (nm)** | | | **Length/width** | |
| --- | --- | --- | --- | --- | --- | --- | --- | --- |
| ***m*_unime_*_r_/***  ***m*_seeds_** | ***L*_n_** | ***L*_w_** | ***L*_w_/*L*_n_** | ***W*_n_** | ***W*_w_** | ***W*_w_/*W*_n_** | ***L*_n_/*W*_n_** | ***L*_w_/*W*_w_** |
| 1 | 108 | 113 | 1.05 | 54 | 60 | 1.11 | 2 | 1.88 |
| 10 | 241 | 245 | 1.02 | 127 | 130 | 1.02 | 1.90 | 1.88 |
| 25 | 361 | 366 | 1.01 | 226 | 230 | 1.02 | 1.6 | 1.59 |
| 40 | 426 | 431 | 1.01 | 279 | 281 | 1.01 | 1.53 | 1.53 |
| 60 | 466 | 470 | 1.01 | 307 | 310 | 1.01 | 1.52 | 1.52 |
| 100 | 501 | 506 | 1.01 | 342 | 345 | 1.01 | 1.46 | 1.47 |

**References**

1. M. C. Arno, M. Inam, Z. Coe, G. Cambridge, L. J. Macdougall, R. Keogh, A. P. Dove, R. K. O'Reilly, *J. Am. Chem. Soc.*, **2017**,139, 16980-16985
